# Identification of anticancer drugs associated to cancer therapy-related cardiac dysfunction: a VigiBase^®^ disproportionality analysis

**DOI:** 10.1093/ehjcvp/pvaf027

**Published:** 2025-04-24

**Authors:** Damien Legallois, Angélique Da Silva, Joachim Alexandre, Paul Milliez, Rémi Sabatier, Katrien Blanchart, Anne-Flore Plane, Jonaz Font, Basile Chrétien, Charles Dolladille

**Affiliations:** Normandie Univ, UNICAEN, INSERM U1086 ANTICIPE, Avenue de la Côte de Nacre, F-14000 Caen, France; Department of Cardiology, Caen-Normandy University Hospital, PICARO Cardio-Oncology Program, Avenue de la Côte de Nacre, F-14000 Caen, France; Normandie Univ, UNICAEN, INSERM U1086 ANTICIPE, Avenue de la Côte de Nacre, F-14000 Caen, France; Department of Pharmacology, Caen-Normandy University Hospital, PICARO Cardio-Oncology Program, Avenue de la Côte de Nacre, F-14000 Caen, France; Department of Medical Oncology, Caen-Normandy University Hospital, PICARO Cardio-Oncology Program, Avenue de la Côte de Nacre, F-14000 Caen, France; Normandie Univ, UNICAEN, INSERM U1086 ANTICIPE, Avenue de la Côte de Nacre, F-14000 Caen, France; Department of Pharmacology, Caen-Normandy University Hospital, PICARO Cardio-Oncology Program, Avenue de la Côte de Nacre, F-14000 Caen, France; Normandie Univ, UNICAEN, INSERM U1237 PhIND, GIP Cyceron, Boulevard Henri Becquerel, F-14000 Caen, France; Department of Cardiology, Caen-Normandy University Hospital, Avenue de la Côte de Nacre, F-14000 Caen, France; Department of Cardiology, Caen-Normandy University Hospital, Avenue de la Côte de Nacre, F-14000 Caen, France; Department of Cardiology, Caen-Normandy University Hospital, Avenue de la Côte de Nacre, F-14000 Caen, France; Department of Cardiology, Caen-Normandy University Hospital, PICARO Cardio-Oncology Program, Avenue de la Côte de Nacre, F-14000 Caen, France; Normandie Univ, UNICAEN, INSERM U1086 ANTICIPE, Avenue de la Côte de Nacre, F-14000 Caen, France; Department of Cardiology, Caen-Normandy University Hospital, Avenue de la Côte de Nacre, F-14000 Caen, France; Department of Advanced Medicine, Nagoya University Hospital, 65 Tsurumai-cho, Showa-ku, Nagoya 466-8560, Japan; Normandie Univ, UNICAEN, INSERM U1086 ANTICIPE, Avenue de la Côte de Nacre, F-14000 Caen, France; Department of Pharmacology, Caen-Normandy University Hospital, PICARO Cardio-Oncology Program, Avenue de la Côte de Nacre, F-14000 Caen, France

**Keywords:** Heart failure, cancer therapy-related cardiac dysfunction, anticancer drugs, pharmacovigilance database

## Abstract

**Aims:**

Therapeutic advancements have significantly enhanced cancer patient survival rates yet concomitantly increased the prevalence of associated toxicities, such as cancer therapy-related cardiac dysfunction (CTRCD), either symptomatic (heart failure) or not. Using the World Health Organization's VigiBase^®^ individual case safety report database, the aim was to establish the association between anticancer drugs and CTRCD reporting.

**Methods and results:**

This study was a disproportionality analysis conducted in VigiBase^®^ from the initial report of any anticancer drug until 29 February 2024. Reporting odds ratios for CTRCD were evaluated using a stepwise selection procedure and multivariable-adjusted analyses. Subsequently, secondary analyses consisted of the description of CTRCD cases associated with the identified anticancer drugs. ClinicalTrials.gov registration number: NCT06268535. Among 36 580 288 database reports, 42 828 CTRCD cases associated with at least one anticancer drug were identified with death reported in 20.6% of cases (8833 CTRCD cases). Primary analysis revealed 25 anticancer drugs significantly associated with CTRCD reporting, with trastuzumab, doxorubicin, and bortezomib exhibiting the strongest associations. Cancer therapy-related cardiac dysfunction reporting was associated with kinase inhibitors, including BCR-ABL inhibitors, ibrutinib, and osimertinib. New signals were identified for trabectedin, clofarabine, fludarabine, entrectinib, gemtuzumab ozogamicin, and anagrelide. In contrast, immune checkpoint inhibitors and most anti-vascular endothelial growth factor therapies showed no association with CTRCD.

**Conclusion:**

This disproportionality study identified 25 anticancer drugs significantly associated with CTRCD reporting, including new signals. It highlights discrepancies compared with drugs recommended for cardiac dysfunction evaluation in the 2022 ESC Guidelines. This underscores the importance of including CTRCD as a safety endpoint in cancer studies.

## Graphical Abstract

**Graphical Abstract:**
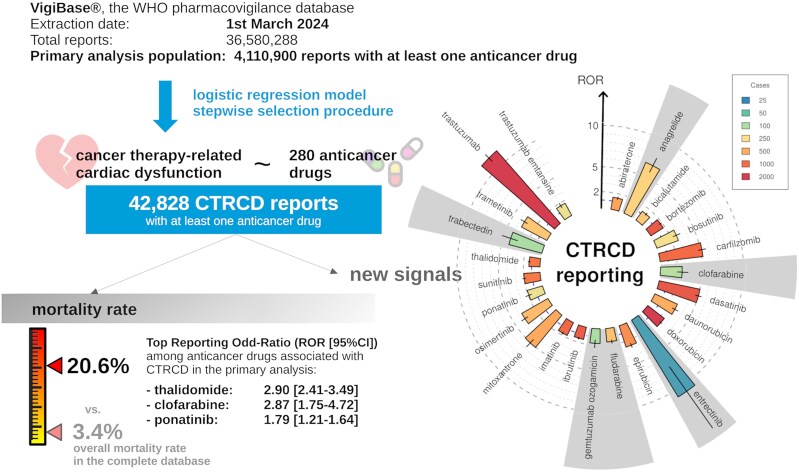
Significant associations between cancer therapy-related cardiac dysfunction cases and anticancer drugs in VigiBase^®^ in the primary analysis population, using the primary analysis (stepwise selection procedure). Only the 25 anticancer drugs significantly associated with CTRCD are displayed among the 280 anticancer drugs. Drugs in grey areas represent new signals. CTRCD, cancer therapy-related cardiac dysfunction and ROR, reporting odd-ratio.

## Introduction

Therapeutic advances have significantly improved the survival of patients with cancer. Given the extension of lifespan and the rapid growth of anticancer drugs indications, these novel therapies are associated with a concomitant increase in the prevalence of adverse drug reactions (ADRs), including cardiovascular ADRs.^1^ Among these ADRs, anticancer drugs can adversely impact cardiac structure and/or function, emerging as asymptomatic cardiac dysfunction or symptomatic heart failure (HF), collectively termed cancer therapy-related cardiac dysfunction (CTRCD).^2^

CTRCD may be caused by both radiotherapy^3^ and anticancer drugs,^4^ with anthracyclines and anti-HER2 agents being the most often described. Identifying a relationship between CTRCD and specific anticancer drugs in clinical trials may be challenging, especially with new anticancer drugs, because (i) most clinical trials are underpowered to adequately assess CTRCD in the cancer population, CTRCD is not always a safety endpoint, (ii) individuals with cardiovascular risk factors, who are at the highest risk of HF, are often excluded from clinical trials,^5^ (iii) there is an overlap between cardiovascular risk factors and cancer risk factors (e.g. smoking) that could contribute to both conditions, and (iv) some instances of CTRCD may manifest shortly after exposure, while others may become apparent only after several years. Access to ’real-world’ data is therefore essential for enhancing our understanding of the relationship between certain anticancer drugs and CTRCD.

The primary objective of this study was to identify anticancer drugs associated with a signal of CTRCD reporting in Vigibase^®^, the World Health Organization (WHO) pharmacovigilance database, using an individual case data analysis. Secondary objectives were to describe the occurrence of cardiogenic shock and death in CTRCD cases associated with anticancer drugs.

## Methods

### Primary objective and analysis

To identify anticancer drugs associated with CTRCD reporting in VigiBase^®^, we performed an observational, retrospective pharmacovigilance study in the WHO pharmacovigilance database (see Supplementary material online, *Methods S1*). VigiBase^®^ allows for disproportionality analysis (also known as case–noncase analysis), a method used to assess whether suspected CTRCD is reported differentially among anticancer drugs. However, it does not establish a formal causal relationship between the drug and the ADR of interest. The Vigibase^®^ extract case level database enables multiple adjustments on concomitant medications, reactions, potential confounding factors and demographic parameters. Details of the analysis and methods are shown in Supplementary material online, *Methods S1*. VigiBase^®^ was the sole data source, and duplicates were removed using the suspected duplicates table included in the dataset. This study complies with READUS-PV guidelines^6^ (see Supplementary material online, *Table S1*), and the protocol was registered on ClinicalTrials.gov (NCT06268535). This study was based on anonymized data from the multinational WHO database, exempting it from the requirement for ethics committee approval in accordance with applicable regulations.

**Figure 1:**
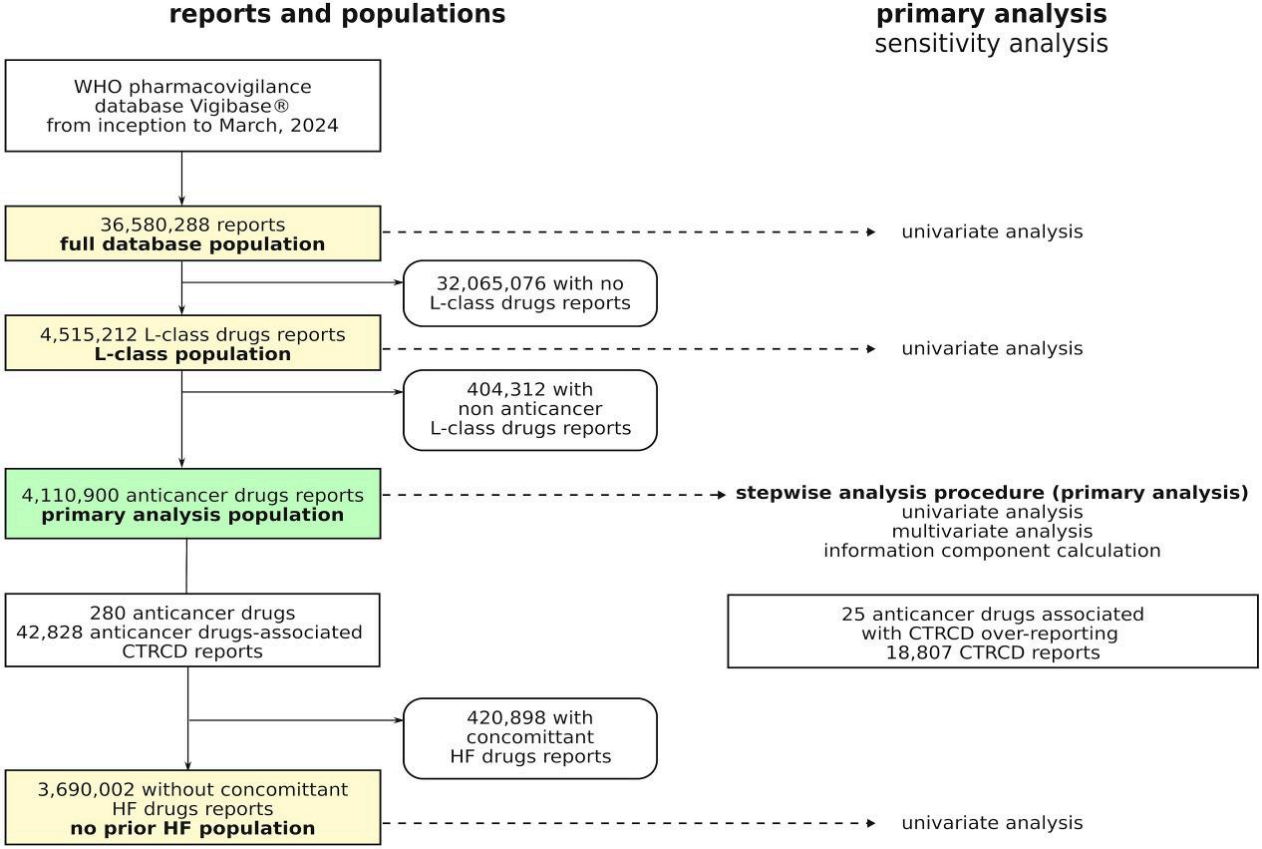
Flow chart of cases selection and analysis in VigiBase^®^. Left: reports and populations. Sensitivity analysis populations include the full database, the L-class (Anatomical and Therapeutic Classification L-class “Antineoplastic and Immunomodulating agents”), and the subgroup with no prior HF (no renin-angiotensin-aldosterone system blockers, diuretics, or beta-blockers). Right: primary analysis in bold, sensitivity analysis in normal font. CTRCD, cancer therapy-related cardiac dysfunction; HF, heart failure.

The study population was restricted to individual case safety reports (ICSRs) involving at least one liable anticancer drug (hereafter the primary analysis population, see *Figure 1*). This strategy is common in disproportionality analysis to limit the indication bias because all patients have received an anticancer drug and hence be likely to have been diagnosed with a cancer.^7^ The comparability between cases and non-cases is improved with this approach. We examined all anticancer drugs labelled by the United States Food and Drug Administration or by the European Medicine Agency prior to October 2023. A total of 280 anticancer drugs were identified (see Supplementary material online, *Table S2*).

Cases were reported with HF and were collected using the ‘Standardized MedDRA query’ (SMQ, from the System Organ Class MedDRA, v26.1): ‘Cardiac failure’ (narrow) from the first report of each anticancer drug until 29 February 2024. The SMQ ’Cardiac Failure’ was used as a proxy for CTRCD as it includes both symptomatic (i.e. HF) and asymptomatic cardiac dysfunction, similar to the CTRCD definition.^2^ The latter term encompasses the broad spectrum of possible presentations of cardiac dysfunction, ranging from mild asymptomatic CTRCD—defined as a LVEF ≥ 50% with a new relative decline in global longitudinal strain of >15% from baseline and/or a new rise in cardiac biomarkers—to very severe symptomatic CTRCD, characterized by HF requiring inotropic support, mechanical circulatory support, or consideration for transplantation. We selected the narrow scope definition, as it is considered more specific in the context of high case numbers^8^ (see Supplementary material online, *Methods S1* for details regarding SMQ terms). Non-cases included all reports without CTRCD.

### Secondary objectives

We described CTRCD cases associated with anticancer drugs extracted from VigiBase^®^, focusing on drugs with a significant reporting odds ratio (ROR) in the primary analysis (all variables are listed in Supplementary material online, *Methods S1*). Univariate analyses assessed the association between anticancer drugs and the seriousness of CTRCD cases. Seriousness was classified as recorded in the database, according to the WHO definition: an ADR associated with death, life-threatening situations, hospitalization, or prolongation of hospitalization, persistent incapacity or disability and with situations judged clinically serious by the physician reporting the case. Cardiogenic shock was identified using the SMQ ‘Shock-associated circulatory or cardiac conditions (excl torsade de pointes)’, and fatal ADRs were defined as CTRCD cases with death as the outcome. Controls were CTRCD reports without cardiogenic shock or death. The results were compared with the IC-OS consensus statement^2^ and the 2022 European Society of Cardiology (ESC) guidelines.^9^ Additionally, disproportionality analysis was used to explore the association between underlying cancer and CTRCD, independent of anticancer drugs. An intersection plot illustrated the overlap of CTRCD reports across multiple drugs.

### Statistical analyses

The primary analysis utilized a logistic regression model with a stepwise selection procedure, retaining adjustment cofactors in the model. The modified Bayesian Information Criterion, which imposes strict penalties for variable selection, was used as the selection criterion.^10^ This conservative approach was chosen to enhance specificity due to the simultaneous study of numerous anticancer drugs. Cofactors included age, sex, relevant concomitant medications, reported cardiovascular conditions, and coprescribed anticancer drugs, as defined in the Supplementary material online, *Methods S1*. These cofactors were consistent across all multivariate analyses.

Six sensitivity analyses were conducted: three in the primary population and three in alternate populations (*Figure 1*). For the primary population, we performed a univariate analysis, a multivariate analysis without the stepwise procedure, and calculated the information component (details in Supplementary material online, *Methods S1*). The last three sensitivity analyses were univariate analyses on alternate populations (*Figure 1*): (i) the L-class population using the Anatomical and Therapeutic Classification ‘Antineoplastic and Immunomodulating agents’, (ii) the full database population without report restrictions, and (iii) a no prior HF population, defined as patients not taking renin-angiotensin-aldosterone system blockers, diuretics, or beta-blockers at the time of reporting, as these concomitant HF drugs served as proxies for a history of HF.

All statistical analyses were performed using R software version 4.3.1 (R Foundation for Statistical Computing, Vienna, Austria) and the most recent version of packages.

## Results

### Comparative study in VigiBase^®^

**Figure 2:**
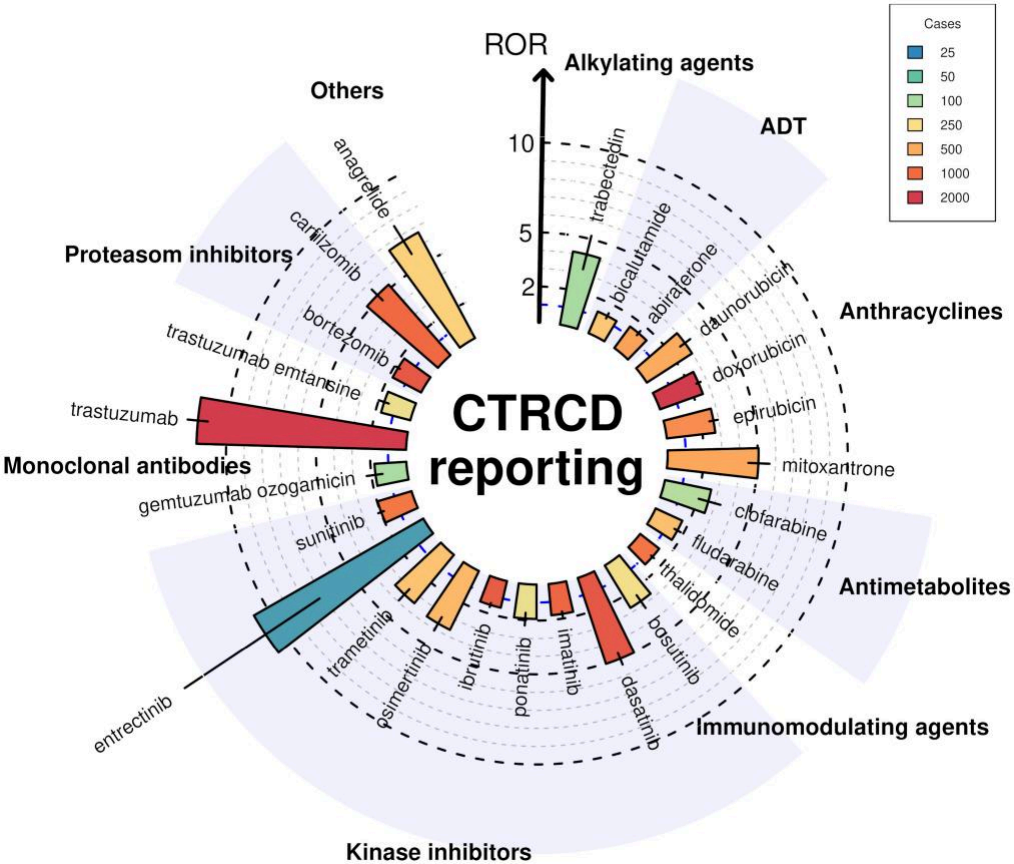
Significant associations between cancer therapy-related cardiac dysfunction cases and anticancer drugs in VigiBase^®^ (primary analysis population). Only 25 anticancer drugs significantly associated with cancer therapy-related cardiac dysfunction, out of 280, are shown. Data were extracted on 1 March 2024, with 42 828 cancer therapy-related cardiac dysfunction cases. CTRCD, cancer therapy-related cardiac dysfunction, ROR, reporting odds ratio.

**Figure 3:**
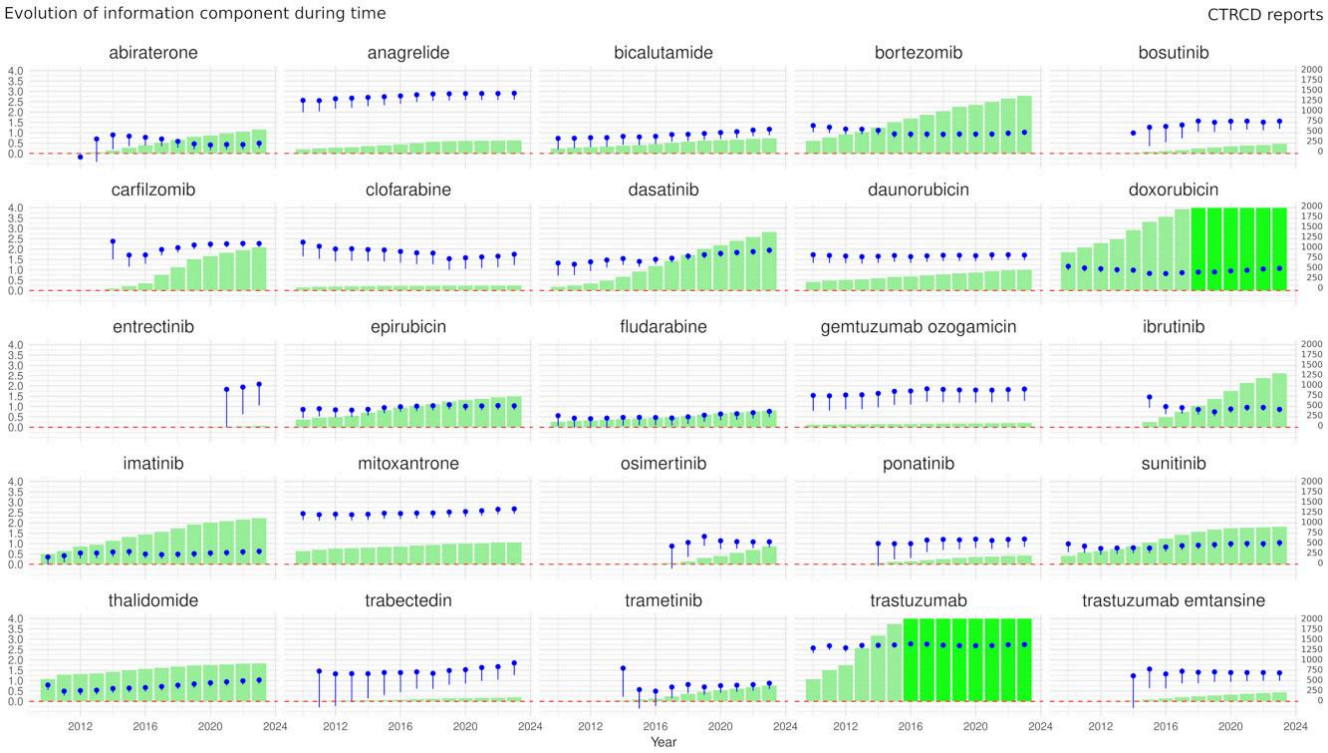
Evolution of the information component over time for the 25 cancer drugs associated with cancer therapy-related cardiac dysfunction in the primary analysis. Dots represent the information component with its lower tail. Bars indicate the number of cases; drugs with more than 2000 cases are shown with a lighter shading.

**Figure 4:**
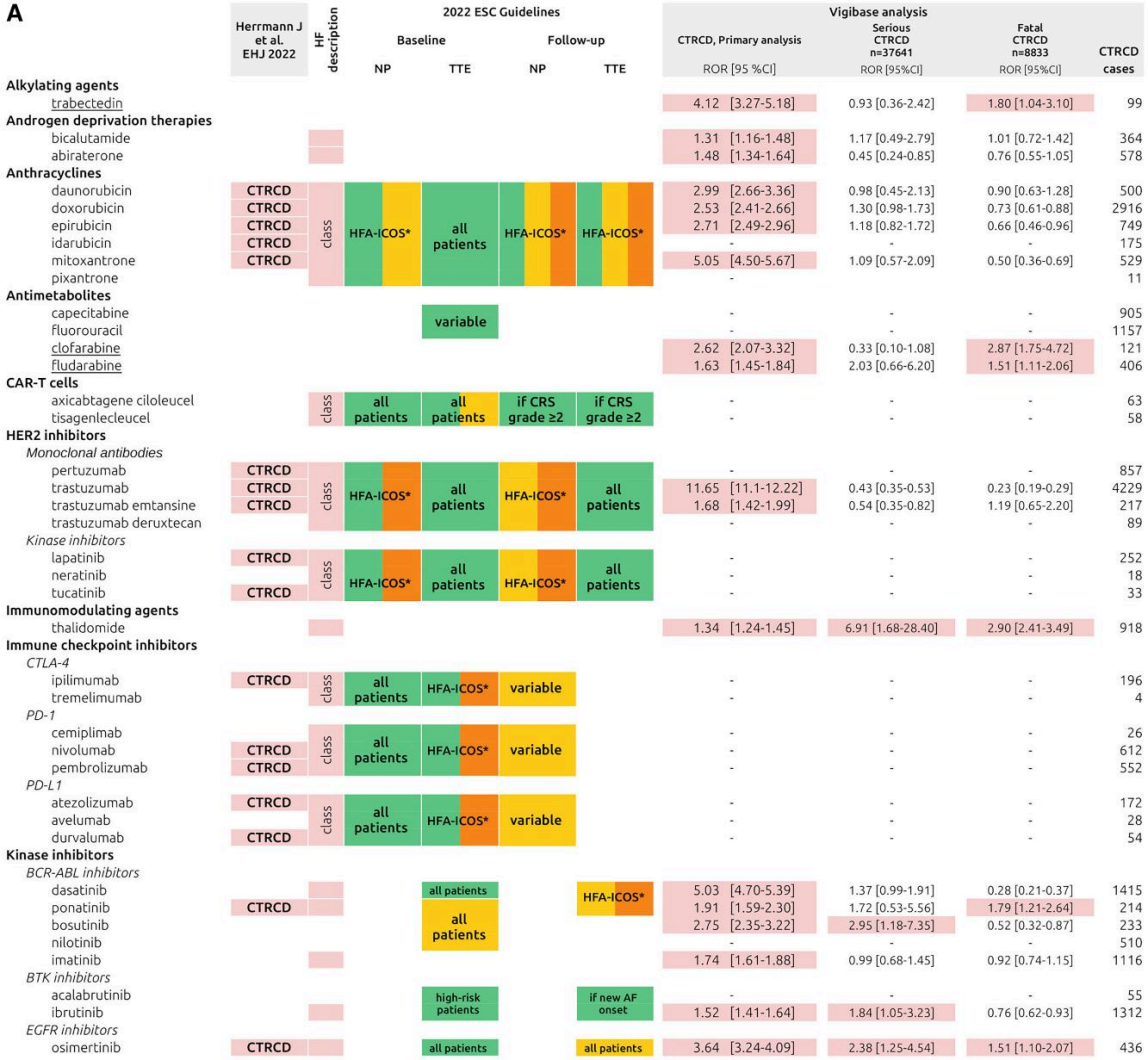

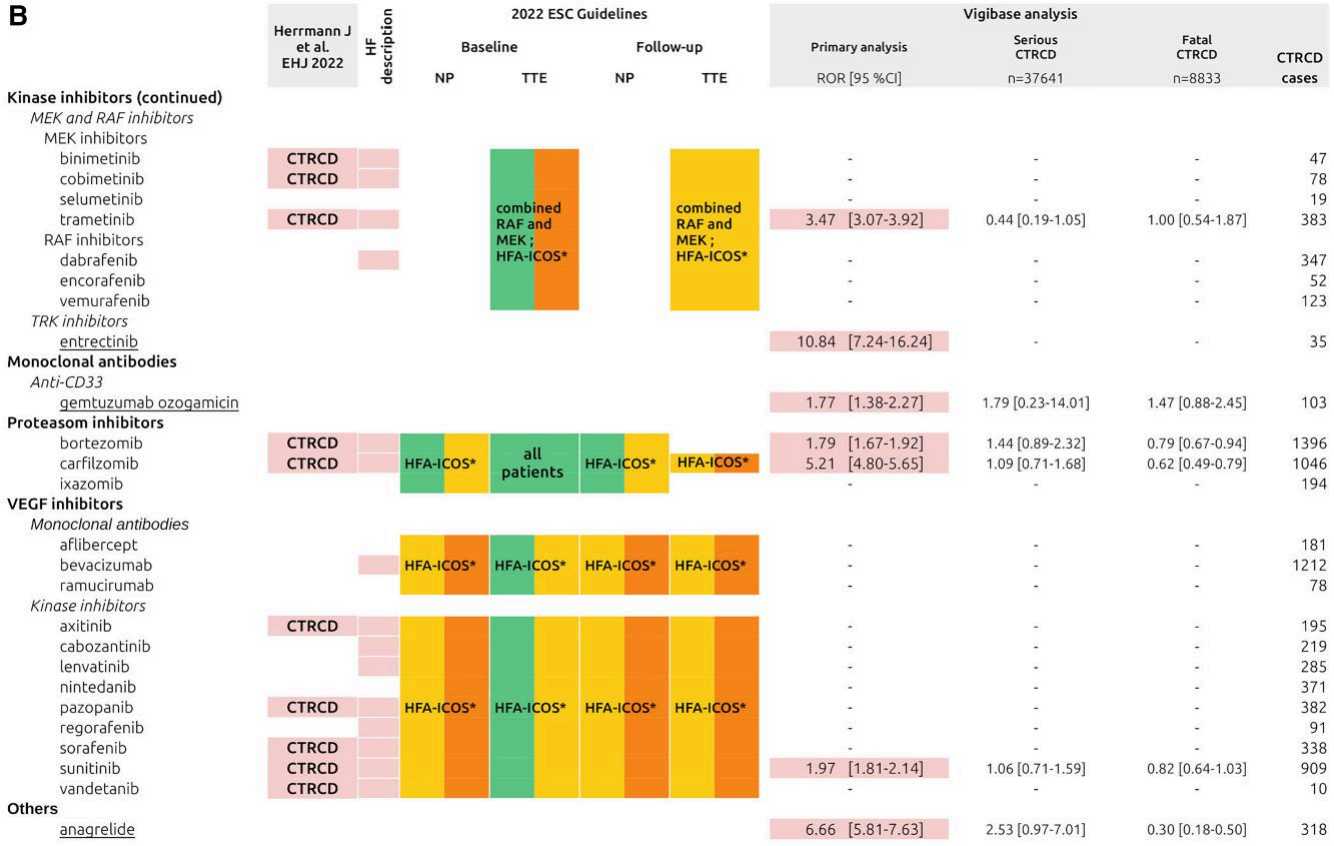
Anticancer drugs associated with cancer therapy-related cardiac dysfunction in the IC-OS consensus statement,^2^ 2022 ESC Guidelines,^9^ or the present analyses (primary, serious cancer therapy-related cardiac dysfunction, and fatal cancer therapy-related cardiac dysfunction). Pink cells: drugs linked to cancer therapy-related cardiac dysfunction in IC-OS. Heart failure column: drugs tied to HF in ESC guidelines. Green, yellow, and orange cells reflect baseline and follow-up recommendations for natriuretic peptides or transthoracic echocardiogram in ESC Guidelines. VigiBase analysis: coloured cells indicate significant associations (overall, serious, and fatal cancer therapy-related cardiac dysfunction). Underlined drugs represent new cancer therapy-related cardiac dysfunction signals not in IC-OS or ESC. CRS, cytokine release syndrome, CTRCD, cancer therapy-related cardiac dysfunction, HFA-ICOS*, indication depends of the HFA-ICOS score of the patient, NP, natriuretic peptides; ROR, reporting odds ratio; TTE, transthoracic echocardiogram.

A total of 4 110 900 reports involving at least one of 280 anticancer drugs were identified (primary analysis population), of which 42 828 (1%) were CTRCD cases (*Figure 1*). Twenty-five anticancer drugs (8.9% of all studied) were associated with CTRCD reporting (*Figure 2*). The strongest association was found for trastuzumab [ROR 11.65; 95% confidence interval, CI (11.10–12.22); 4229 CTRCD cases]. Among targeted anticancer drugs, CTRCD was associated with several kinase inhibitors (KIs), including BCR-ABL inhibitors with dasatinib showing the highest ROR [5.03; 95% CI (4.70–5.39); 1415 CTRCD cases]. The BTK inhibitor ibrutinib [ROR: 1.52; 95% CI (1.41–1.64); 1312 CTRCD cases], the EGFR inhibitor osimertinib [ROR: 3.64; 95% CI (3.24–4.09); 436 CTRCD cases], the MEK inhibitors trametinib, entrectinib, and the multitarget KI sunitinib were also associated with CTRCD reporting. Among monoclonal antibodies, besides trastuzumab and trastuzumab emtansine, only gemtuzumab ozogamicin was associated with CTRCD. Additionally, several older anticancer drugs were associated with CTRCD, including trabectedin, androgen deprivation therapies (ADT) (bicalutamide and abiraterone), antimetabolites (clofarabine and fludarabine), thalidomide, anagrelide [ROR 6.66; 95% CI (5.81–7.63); 318 CTRCD reports], and proteasome inhibitors bortezomib and carfilzomib. The information component analysis over time showed relative stability for the 25 anticancer drugs significantly associated with CTRCD (*Figure 3*). Overall, our primary analysis identified ten anticancer drugs either not previously associated with CTRCD or without specific recommendations in the IC-OS consensus statement^2^ or ESC 2022 guidelines^9^ (*Figure 4* and *Graphical abstract*): abiraterone, anagrelide, bicalutamide, clofarabine, entrectinib, fludarabine, gemtuzumab ozogamicin, imatinib, thalidomide, and trabectedin. Detailed results of disproportionality analyses for all 280 anticancer drugs, including sensitivity analyses, are presented in Supplementary material online, *Table S2*.

Out of all 42 828 CTRCD cases, 87.9% were categorized as serious (37 641 CTRCD cases), with death reported in 20.6% of cases (8833 CTRCD cases), as compared with 11 709 508 serious events and 1 234 887 deaths in the overall database (36 580 288 cases, 32.0% and 3.4%, respectively). Among the anticancer drugs associated with CTRCD reporting in primary analysis, trabectedin, clofarabine, fludarabine, thalidomide, ponatinib, and osimertinib were associated with an over-reporting of fatal outcome compared with other drugs (*Figure 4* and Supplementary material online, *Table S3*).

### Descriptive cohort in Vigibase^®^

**Figure 5:**
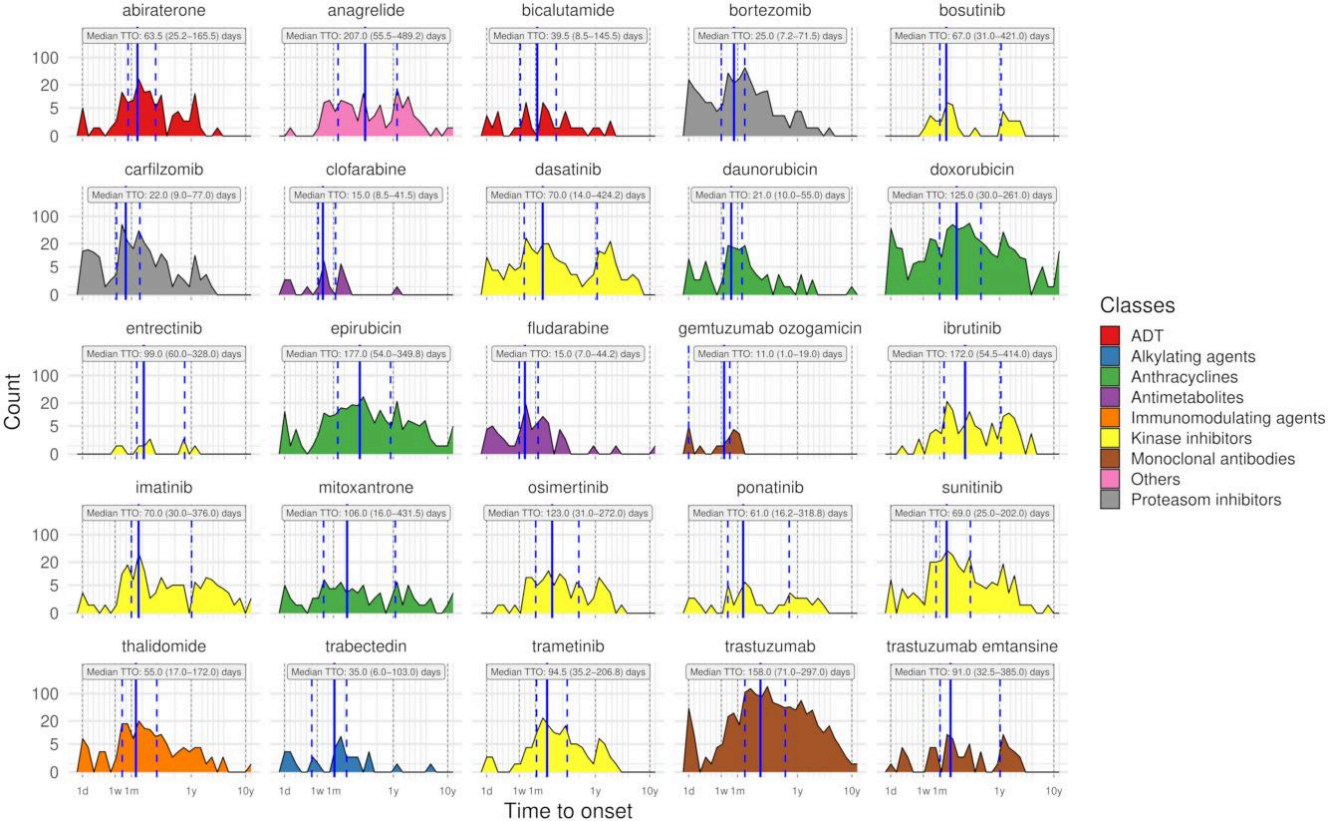
Time to cancer therapy-related cardiac dysfunction onset from treatment initiation for the 25 drugs associated with cancer therapy-related cardiac dysfunction reporting in the primary analysis. Time to onset are displayed as median and interquartile values, in days. ADT, androgen deprivation therapies; TTO, time to onset.

The associations between CTRCD cases and patient demographics, concurrent medications, and reactions are shown in Supplementary material online, *Figure S1*. The 25 anticancer drugs associated with CTRCD in VigiBase^®^ account for 18 807 cases. Supplementary material online, *Table S4* and Supplementary material online, *Table S5* provide details on concurrent medications, diseases, and demographics. The median time to onset (TTO) for the 25 drugs was 90 days (interquartile range: 23–252) across 4530 reports (24.1%, *Figure 5*). Cancer therapy-related cardiac dysfunction was more frequently reported soon after proteasome inhibitor exposure [bortezomib: median TTO: 25 days (interquartile range: 7.2–71.5), carfilzomib: median TTO: 22 days (interquartile range: 9.0–77.0)]. Time to onset was longer with KI treatment [median: 83 days (interquartile range: 27–299)]. Supplementary material online, *Table S6* shows additional TTO values. Common anticancer drug coprescriptions in CTRCD cases are shown in Supplementary material online, *Figure S2*. Supplementary material online, *Table S7* lists cancer indications for the 25 drugs. Exploratory analysis (44.7% of cases) suggests CTRCD is linked to soft tissue, breast, kidney cancers, and haematologic malignancies (see Supplementary material online, *Figure S3*).

## Discussion

Among the largest collection of CTRCD cases to date, we identified 25 anticancer drugs associated with CTRCD in the WHO pharmacovigilance database, Vigibase^®^. Of these anticancer drugs, 10 were either previously unknown or, although documented, lacked specific recommendations in current guidelines. These findings enhance the understanding of CTRCD-anticancer drug associations and underscore the need for systematic evaluation of CTRCD in oncologic randomized clinical trials.

CTRCD, especially HF, raises significant concerns for cancer patients. Studies show that HF patients have worse outcomes when cancer is present,^11^ while severe cardiotoxicity during or after cancer treatment increases all-cause mortality. In the CARDIOTOX Registry,^12^ 865 patients on moderate or high-risk anticancer therapies were enrolled. Those with left ventricular ejection fraction ≤40% or symptomatic HF had a mortality rate of 22.9 per 100 patient-years, compared with 2.3 per 100 patient-years in those without severe cardiotoxicity. These findings, consistent with our analysis showing over 20% mortality in cancer patients, emphasize the need for better CTRCD risk assessment. Our findings indicate associations between CTRCD and several anticancer drugs lacking recommendations for treatment surveillance and ADTs serve as prime examples due to their widespread use. With prostate cancer being the second most common cancer globally, with an estimated 1.4 million new cases in 2020, ADTs are widely regarded as the cornerstone of advanced prostate cancer treatment.^13^ In the present work, we found an association between CTRCD and bicalutamide and abiraterone. Previous studies have demonstrated an association between certain antiandrogenic drugs and HF^14^ and the American Heart Association recently published a statement about the impact of hormonal therapies including ADTs on the cardiovascular system.^15^ A 2017 pharmacovigilance study^16^ reported an over-reporting of HF with abiraterone, aligning with our analysis showing a significant signal from 2014 (*Figure 3*). Abiraterone, a CYP17 inhibitor, elevates steroidal compound levels, leading to secondary mineralocorticoid excess, possibly explaining its higher HF risk compared with enzalutamide, a drug with similar indications.^16^ Screening and follow-up for ADT-treated prostate cancer patients may be difficult, but focus should be placed on high-risk groups (history of HF, hypertension, atrial fibrillation, or use of ADT medications with hypermineralocorticoid properties). Baseline risk assessment and close monitoring, especially in the first 6 months, may be advisable in these populations.^17^

In our analysis, aside from trastuzumab, entrectinib showed the strongest association with CTRCD [ROR 10.84; 95% CI (7.24–16.24)], despite no specific cardiovascular assessment recommendations in the 2022 ESC Guidelines.^9^ Entrectinib, a multikinase inhibitor, was evaluated in a pooled safety analysis of 224 ROS1 fusion-positive non-small-cell lung cancer patients.^18^ Left ventricular ejection fraction reduction occurred in 1.3% of patients, and HF in 2.7%, mostly grade 3 or 4. The Food and Drug Administration recommends left ventricular ejection fraction assessment before starting entrectinib in patients with symptoms or HF risk factors, and monitoring for HF symptoms.^19^ This is based on the occurrence of HF in 3.4% of patients across trials, despite excluding those with symptomatic HF or prior coronary events. Additionally, no baseline cardiac function assessments or routine monitoring were conducted beyond electrocardiograms. Heart failure resolved in 50% of affected patients after discontinuing entrectinib and initiating appropriate treatment. The median TTO was 2 months, consistent with our findings. Trabectedin, an alkylating agent for soft tissue sarcoma, has recently been identified as a potential cause of CTRCD.^20^ However, the database does not provide exhaustive information on prior exposure to anticancer drugs. Since trabectedin is used in the treatment of sarcoma, it is likely that some patients may have been treated with anthracyclines years, or even decades, earlier. Anagrelide, used for essential thrombocythemia, has been associated with palpitations, oedema, hypertension, and myocardial infarction,^21^ along with cases of reduced left ventricular ejection fraction. Our results should be interpreted with caution, as thrombocythemia may increase the risk of clotting, which could indirectly lead to HF. Among proteasome inhibitors, carfilzomib showed the strongest association with CTRCD, as expected.^22^ Bortezomib has a safer cardiovascular toxicity profile, but cardiovascular ADRs, including HF, occurred with both drugs in multiple myeloma patients.^23^ Carfilzomib therapy posed a higher risk than bortezomib (HR: 3.0; 95% CI: 1.1–8.4; *P* = 0.04), with most HF events occurring within the first 3 months, consistent with our TTO analysis.

Immune checkpoint inhibitors (ICIs) have transformed cancer treatment, being used for melanoma, lung, renal, and other cancers, with 36.1–38.5% of US cancer patients eligible for ICI therapy in 2019.^24^ Baseline cardiovascular evaluation, including biomarkers and echocardiograms, is recommended in the 2022 ESC Guidelines^9^ for high-risk patients on ICIs. However, our analysis did not find a significant association between CTRCD and ICIs. Though ICIs have been associated with myocarditis^25^ and HF^26^ in prior studies, our larger dataset and statistical methods likely better accounted for confounding factors. Heart failure may have been a common manifestation of ICI-related myocarditis, which was less recognized in the past years. Moreover, myocarditis remains a rare condition, and the incidence of HF outside this context is relatively low, which may explain why ICIs were not associated with CTRCD in the present study. A safety meta-analysis of randomized clinical trials estimated HF incidence in ICI-treated patients at 8.7 per 1000.^26^ In our analysis, nivolumab, pembrolizumab, and ipilimumab had low CTRCD proportions (0.8%, 0.9%, and 0.6% of reports, respectively), consistent with studies showing a low HF incidence with ICI use.^27^ Another explanation may be that, in the early years of ICIs use, myocarditis was not yet well recognized and was often coded as HF. According to MedDRA guidelines, underlying conditions should not be coded when a definitive diagnosis is available. As myocarditis is now a well-defined entity, such cases are likely no longer coded as HF.

Our study revealed some discrepancies with existing literature. Aside from sunitinib, we found no significant association between CTRCD and anti-vascular endothelial growth factor (VEGF) therapies. This could be due to competitive and notoriety biases from the early 2000s when these drugs were introduced. Hypertension is a common ADR with anti-VEGF therapies,^9,28^ and heightened awareness may have led to improved blood pressure monitoring and management using angiotensin system inhibitors. These inhibitors have been associated with better outcomes in metastatic renal cell carcinoma patients on anti-VEGF therapies,^29,30^ potentially reducing HF incidence and underscoring the value of preventing and managing cardiovascular risks induced by these anticancer drugs.

We acknowledge several limitations in this pharmacovigilance study. While such databases are essential for real-world safety assessments, they often include non-homogeneous, incomplete reports. Most data in VigiBase came from North America and Western Europe. Also, we did not compute sex and gender-based analyses. As a consequence, generalization should be made with caution. Disproportionality analyses have inherent biases, so results should be interpreted cautiously. These analyses compare ADR-drug relationships but lack medication sales data, making it impossible to infer ADR incidence for specific drugs. Additionally, certain patient-related factors (e.g. concomitant conditions) or cancer-related factors may have confounded our results, as discussed earlier in relation to the association between anagrelide and CTRCD reporting. Any ADR deemed clinically serious by the reporting physician was classified as ‘serious’, which may have contributed to the high proportion of serious CTRCD cases observed during anticancer therapy. While we performed a multivariate analysis to mitigate these influences, it is possible that some confounding factors were not identified. As previously discussed, drawing definitive conclusions about the association between a specific anticancer drug and CTRCD reporting can be challenging, especially when patients may have received another anticancer drug associated with a known long delay in the risk of HF. Our use of the narrow SMQ term ‘Cardiac failure’ does not distinguish between altered and preserved left ventricular ejection fraction. However, this approach aligns with the ESC Guidelines,^9^ even though we acknowledge that a complete overlap between SMQ and CTRCD definitions is not expected. Overall, disproportionality analyses have inherent biases that may necessitate a cautious interpretation of their results. This type of pharmacovigilance study serves as an indicator of a possible association between a drug and an adverse effect, but multiple approaches (e.g. clinical trials or translational science) are warranted to definitively address the relationship between a drug and the risk of an ADR.

Using Vigibase^®^, we identified 25 anticancer drugs associated with CTRCD. New signals emerged for some anticancer drugs while others, such as bicalutamide and abiraterone, were already linked to cardiac dysfunction but lacked cardiovascular assessment guidelines. About one-fifth of the reports associated with cardiac dysfunction involved death, underscoring the poor prognosis of cardiac dysfunction in cancer therapy. Multiple approaches, including safety data from randomized clinical trials and real-world databases, are needed to better stratify CTRCD risk in cancer patients.

## Supplementary Material

pvaf027_Supplementary_Data

## Data Availability

The datasets generated and/or analysed during the current study are not publicly available as data were extracted from VigiBase^®^, the WHO global database of individual case safety reports, made available by the Uppsala Monitoring Centre (Uppsala, Sweden). Restrictions apply to the availability of these data, which were used under license for the current study, and so are not publicly available. Requests can be made to the corresponding author who will submit it to the Uppsala Monitoring Centre (Uppsala, Sweden, https://who-umc.org/vigibase/) to obtain its permission.
