# Supplementary material for: Identification of anticancer drugs associated to cancer therapy-related cardiac dysfunction: a VigiBase® disproportionality analysis
Source: Eur Heart J Cardiovasc Pharmacother. 2025 Apr 24;11(5):459–68. doi: 10.1093/ehjcvp/pvaf027 (PMC12342995; doi:10.1093/ehjcvp/pvaf027)

Identification of Anticancer Drugs Associated to Cancer Therapy-Related Cardiac Dysfunction: A VigiBase® Disproportionality Analysis

Supplemental Data. Summary

Supplemental Methods 1 .... page 2

Supplemental Table 1. The READUS-PV checklist .... page 5

Supplemental Table 2. Reporting Odds Ratio and their confidence interval of the 280 anticancer drugs with cancer therapy-related cardiac dysfunction in VigiBase .... page 7

Supplemental Table 3. Reporting Odds Ratio and their confidence interval of serious events, cardiogenic shock and death of the anticancer drugs associated with cancer therapy-related cardiac dysfunction in either primary analysis or multivariate sensitivity analysis in the primary population in VigiBase .... page 14

Supplemental Table 4. Characteristics of cancer therapy-related cardiac dysfunction (CTRCD) cases of the anticancer drugs associated with CTRCD in either primary analysis or multivariate sensitivity analysis in Vigibase .... page 16

Supplemental Table 5. Characteristics of concomitant medications in cases of cancer therapy-related cardiac dysfunction (CTRCD) associated with the anticancer drugs associated to CTRCD overreporting in Vigibase® in either primary analysis or sensitivity multivariate .... page 18

Supplemental Table 6. Time to onset from treatment initiation for the anticancer drugs associated with cancer therapy-related cardiac dysfunction overreporting in Vigibase® in our multivariate sensibility analysis .... page 20

Supplemental Table 7. Cancer indication for the anticancer drugs associated with cancer therapy-related cardiac dysfunction overreporting in VigiBase® in primary analysis .... page 21

Supplemental Figure 1. Associations between CTRCD reporting and concurrent medications, concurrent reactions, and demographic characteristics of patients in VigiBase® in the primary analysis population .... page 23

Supplemental Figure 2. Overview of the most prevalent anticancer drug regimens among cancer therapy-related cardiac dysfunction (CTRCD) reports in Vigibase® .... page 24

Supplemental Figure 3. Associations between cancer therapy-related cardiac dysfunction cases and indications for cancer therapies in VigiBase® within the primary analysis population .... page 25

Supplementary Data 1 – Supplementary methods

Vigibase® and Vigibase® extract case level structure.

VigiBase® is managed by the Uppsala Monitoring Centre (Uppsala, Sweden) and contains more than 36 million individual case safety reports (ICSRs) received from at least 130 member countries since 1967. ICSRs originate from different sources, including healthcare professionals, patients, and pharmaceutical companies, and are generally notified postmarketing. ICSRs include administrative information (country, type of report, qualification of reporter), patient data (age, sex), date of onset of reaction(s) and the nature of the adverse drug reaction (ADR) using the latest version of MedDRA (Medical Dictionary for Regulatory Activities) terms. Drug(s) involved (name, drug start and stop dates, time to onset, indication, dose) are also indicated. Drugs are coded using the WHO drug dictionary, covering over 150,000 medicines and vaccines. Information included in VigiBase® comes from a variety of sources, and the probability that the suspected adverse effect is drug-related is not the same in all cases.

The Vigibase® extract case level data is a relational database. Its structure allows for multiple adjustments on potential confounding factors. The demographic table includes data on the year of reporting, the patient age and sex, and the country of reporting. The outcome table provides information on seriousness and death. The ADR table includes data on each ADR of ICSRs, coded with MedDRA codes. The medications table includes data on each drug of ICSRs, coded with medicinal product Id (drug name, dose, route of administration, frequency, dechallenge and rechallenge). The time to onset is only reported for cases with complete dates available and is precalculated for each pair of medication and reaction in ICSRs in a link table. Indication of the medications is provided in an indication table.

Description of the pharmacovigilance cohort in VigiBase®.

For each report, age (categorized into the following categories: "<18yr", "18-45yr', "45-64yr", "65-74yr" and "75yr"), sex, drug indication, time from anticancer drug initiation to CTRCD occurrence (here after time to onset (TTO)), outcome, concurrent medications and ADRs were collected (see Supplementary Data for details). To identify concurrent medications in heart failure (HF) ICSRs, we used the Anatomical and Therapeutic Classification (ATC) of drugs. The concurrent medications of interest were as follows: ATC classes H02 "Corticosteroids for systemic use", C09 "Agents acting on the renin-angiotensin system", C03 "Diuretics", and C07 "Beta blocking agents".

To identify potential concurrent diseases or conditions associated with HF reporting, we analyzed the co-reported conditions within the ICSR using Standardized MedDRA queries (SMQs) or System Organ Class (SOC) terms. SMQs have been developed to refine the identification of diseases applicable to pharmacovigilance databases using predefined sets of terms of special interest [S1]. There is a broad and narrow definition of each SMQ, that is considered more sensitive and more specific, respectively [S1]. The concurrent diseases or conditions were as follows: SMQ narrow "Hypertension" for hypertension, SMQ narrow "Embolic and thrombotic events, venous" for both pulmonary emolism and/or deep venous thrombosis, SMQ narrow "Myocardial infarction" for acute coronary syndrome, SMQ narrow "Supraventricular tachyarrhythmias" for atrial fibrillation, atrial tachycardia or atrial flutter, SMQ narrow "Ventricular tachyarrhythmias" for ventricular tachycardia, SMQ narrow "Torsade de pointes/QT prolongation" for torsade de pointes or QT prolongation, SMQ narrow "Shock-associated circulatory or cardiac conditions (excl torsade de pointes)" for cardiogenic shock, SMQ narrow "Hyperthyroidism" for hyperthyroidism, high level group SOC term "Cardiac valve disorders" for cardiac valve disorders, high level SOC term "Noninfectious myocarditis" for myocarditis and, high level SOC term "Cardiac conduction disorders" for cardiac conduction disorders. An ICSR was classified as serious as recorded in the database. Cardiogenic shock was defined as HF reported using the SMQ narrow "Shock-associated circulatory or cardiac conditions (excl torsade de pointes)".

Definition of the SMQ narrow "Cardiac failure" in VigiBase®.

The definition of the SMQ narrow "Cardiac failure" includes the following perferred terms :

- Cor pulmonale acute
- Acute pulmonary oedema
- Cardiac failure acute

- Cardiac asthma
- Cardiac failure congestive
- Cardiac failure
- Cardiac failure chronic
- Cardiac failure high output
- Left ventricular failure
- Right ventricular failure
- Cardiogenic shock
- Cor pulmonale chronic
- Congestive hepatopathy
- Cor pulmonale
- Pulmonary oedema
- Ejection fraction decreased
- Low cardiac output syndrome
- Cardiopulmonary failure
- Neonatal cardiac failure
- Ventricular failure
- Pulmonary oedema neonatal
- Hepatojugular reflux
- Acute left ventricular failure
- Acute right ventricular failure
- Chronic left ventricular failure
- Chronic right ventricular failure
- Cardiorenal syndrome
- Obstructive shock
- Right ventricular ejection fraction decreased
- Radiation associated cardiac failure
- Cardiohepatic syndrome

We did not studied HF according to left ventricular ejection fraction because this data was only available for a small portion of the dataset, making it impossible to draw any meaningful conclusions about the patterns of left ventricular ejection fraction associated with HF reporting, even using missing data imputation procedures.

Statistical analyses.

Disproportionality analysis compares the proportion of a selected specific ADR reported for a single drug or group of drugs with the proportion of the same ADR for a control group of drugs in an ICSR population (e.g., full database population or any subset of it). The denominator in these analyses is the total number of ADRs reported for each group of drugs. If the proportion of patients exposed to a specific drug is greater in the selected ADR group (cases) than in patients without this ADR (noncases), there is a disproportionality association between the ADR and the drug. The result of this comparison can be expressed as a ROR, computed by a χ^2 test (for univariable analysis) or a logistic regression model with Wald test when multivariable adjustment on other covariates (such as age) is required. The reporting odd-ratio (ROR) estimate has been shown to be most reliable in the presence of at least 3 to 5 cases for the ADR of interest (herein, CTRCD) [S4]. Therefore, we computed the ROR for anticancer drugs including at least 5 CTRCD cases. The time window period was restricted to the period of notification of the anticancer drug group.

The information component (IC) is an automated indicator based on a Bayesian confidence propagation neural network. It compares the observed and expected number of reports for drug-ADR pairs. The IC_{025} is the lower end of the 95% credibility interval for the IC. A positive value of IC_{025} is deemed significant for detecting safety signals (IC_{025}>0) [S2,S3].

References

[S1] Mozzicato P. Standardised MedDRA Queries. Drug Safety. 2007;30(7):617–619.

[S2] Caster O, Juhlin K, Watson S, Norén GN. Improved Statistical Signal Detection in Pharmacovigilance by Combining Multiple Strength-of-Evidence Aspects in vigiRank. Drug Safety. 2014;37(8):617–628.

[S3] Bate A, Lindquist M, Edwards IR, Olsson S, Orre R, Lansner A, et al. A Bayesian neural network method for adverse drug reaction signal generation. Eur J Clin Pharmacol. 1998;54(4):315–321.

[S4] Puijenbroek EP van, Bate A, Leufkens HGM, Lindquist M, Orre R, Egberts ACG. A comparison of measures of disproportionality for signal detection in spontaneous reporting systems for adverse drug reactions. Pharmacoepidemiol Drug Saf. 2002;11(1):3-10.

Supplemental Table 1. The READUS-PV checklist

Section and topic	Item #	Checklist item
Title		
	1a	If disproportionality analyses are a prominent component of the published study, the study should be identified as a "disproportionality analysis." The type of data and name of the database(s) should be specified.<br>Title : Identification of Anticancer Drugs Associated with Cancer Therapy-Related Cardiac Dysfunction: A Disproportionality Analysis of the Pharmacovigilance Database VigiBase®
	1b	Report the name of adverse event(s) and/or drug(s) under study, when applicable<br>The terms « anticancer drugs » and « cancer therapy-related cardiac dysfunction » are reported in the title.
Introduction		
Background	2a	Describe the drug(s) and its utilization, the nature of the adverse event(s) under study and its frequency, and the existing knowledge on the drug–event combination.<br>Done in the « Introduction » section.
	2b	Specify the rationale for performing the analysis, e.g., as part of routine pharmacovigilance, to investigate an overall safety profile, or to assess a pre-specifed hypothesis.<br>The rationale for this study is to identify anticancer drugs associated with signals of cancer therapy-related cardiac dysfunction (CTRCD), given its frequency and severity as an adverse event in patients treated with these drugs. See « Introduction » section.
	2c	Explain why individual case safety report databases and disproportionality analysis are suitable to fill the knowledge gap.<br>Done in the « Introduction » section.
Objectives	3	State specific objectives, identifying the adverse event(s), the drug(s), and the reference group, including any pre-specifed hypothesis, if applicable.<br>Done in the « Primary objective and analysis » subsection.
Methods		
Study design	4a	Identify the study (i.e., "disproportionality analysis") and the type of data used (e.g., "individual case safety reports").<br>Done in the « Primary objective and analysis » subsection.
	4b	Provide an outline of the entire study design, including primary and sensitivity analyses performed, and other designs such as case-by-case analysis or literature review<br>Done in the « Primary objective and analysis » and in the « Secondary objectives » subsections.
Data description, access, and pre-processing	5a	Specify the name of the database(s), the database(s) custodian, and the coverage. Specify the type/number of drugs included within the database and the thesaurus, taxonomies, or ontologies used for coding drugs and events.<br>Done in the « Primary objective and analysis » subsection and in the Supplementary Data 1.
	5b	Specify the extraction dates and describe and justify all choices used for data pre-processing, including anydata transformation or exclusion, if appropriate.<br>Done in the « Primary objective and analysis » subsection.<br>We utilized the suspected duplicates table available in the database.
Variables defnition	6a	Describe the study population, including any restriction<br>Done in the « Primary objective and analysis » subsection.
	6b	Describe the nature and the meaning of key variables assessed in the work<br>Done in the « Variables » subsection.
	6c	Specify and justify any grouping of drugs or events. For drugs, specify and justify whether active ingredients/trade names/salts were considered and/or the selected role.<br>The « Primary objective and analysis » section explains how the Standardized MedDRA querry « Cardiac Failure » is a proxy for cancer therapy-related cardiac dysfunction.
	6d	Describe any additional data source used, the type of data, and how they interact with individual case safety reports.<br>It is stated in the « Primary objective and analysis ».
Statistical methods	7a	Present any descriptive analysis performed, specifying variables investigated, statistical tests, and signifcance thresholds.<br>Done in the « Statistical analyses » subsection.
	7b	Present any descriptive analysis performed, specifying variables investigated, statistical tests, and signifcance thresholds.

Section and topic	Item #	Checklist item
		Done in the « Statistical analyses » subsection.
	7c	Clearly describe any sensitivity analysis and any tool to control confounding, including any restriction, subgroup, stratifcation, adjustment, or interaction<br>Done in the « Statistical analyses » subsection.
	7d	Specify the variables and methods used for the case-by-case analysis, including any algorithm or criteria used to assess causality, if performed.<br>Done in the « Variables » and « Statistical analyses » subsections.
	7e	Specify any statistical methods used for other data sources.<br>N/A. There is no other data source than VigiBase (see item 6d)
Results		
Participants	8a	Specify the number of individual case safety reports included at each stage, including reasons for exclusion<br>See Figure 1.
	8b	Provide key demographic and clinical characteristics of cases, if possible comparing cases with any appropriate reference group<br>See Figure 4.
Disproportionality analysis	9	Present all results including confdence intervals. Present also results of sensitivity analyses, if performed.<br>Done in the «Comparative study in VigiBase®» subsection.
Case-by-case analysis	10	Present the case-by-case analysis of key variables. Present the causality assessment, if applicable<br>Done in the «Descriptive cohort in Vigibase®.» subsection.
Discussion		
Key results	11	Discuss key results with reference to study objectives and contextualize them within the current literature and other consulted sources. Clearly discriminate between expected reactions and emerging safety signals.<br>Done in the «Discussion» section.
External validity	12a	Discuss the external validity of the results to the general population<br>Done in the «Discussion» section: «Most data in VigiBase came from North America and Western Europe, so generalization should be done cautiously.»
	12b	Discuss the potential relevance of results in clinical practice<br>Done in the «Discussion» section.
	12c	Propose further study designs if applicable<br>Done in the «Discussion» section.
Limitations	13	Present general limitations, making clear that disproportionality analysis alone cannot prove causation or measure incidence, and specifc limitations, including confounding and reporting bias and eforts to mitigate them.<br>Done in the last paragraph of the «Discussion» section : « Limitations »
Declarations	14a	Provide the source of funding/sponsorship and the role of the funders/sponsors for the present study and for any original study on which the present article is based.<br>Done in the « Funding » section.
	14b	Clearly identify potential commercial and intellectual conficts of interest (e.g., link to any drug/event investigated, whether fnancial, legal action, or software used).<br>Done in the « Disclosure of interest » section.
	14c	Declare any institutional approval needed or granted in the investigation.<br>No institutional approval was needed.
	14d	Include a statement on data availability, code availability (including the version of the statistical software used), and protocol registration.<br>Done in the « Data availability statement » section.

Supplemental Table 2

Reporting Odds Ratio and their confidence interval of the 280 anticancer drugs with cancer therapy-related cardiac dysfunction (CTRCD) in VigiBase®. Primary analysis is a stepwise selection procedure, where adjustment cofactors were maintained in the model. Sensitivity analysis: multivariate and univariate analysis in the primary population, univariate analysis in the L-class population, univariate analysis in the full database population, univariate analysis in the no prior HF population. Date of extraction was March, 1st, 2024. ALK: anaplastic lymphoma kinase, BCL2: B-Cell Lymphoma 2, BCR-ABL: Breakpoint Cluster Region – Abelson, BTK: Bruton Tyrosin Kinase, CDK: cyclin-dependent kinase, CI: confidence interval, CTLA4: cytotoxic T-lymphocyte antigen 4, CYP17: 17-alpha-hydroxylase/C17,20 lyase, EGFR: endothelial growth factor receptor, FLT3: fms-like tyrosin kinase 3, GnRH: gonadotrophin releasing hormone, HER2: human epidermal growth factor 2, HF: heart failure, IC025: lower-end of the Information Component confidence interval, MEK: mitogen-activated extracellular kinase, mTOR: mechanistic target of rapamycin, PDGFRa: platelet-derived growth factor receptor alpha, PI3K: phosphatidylinositol 3-Kinase, RAF: Rapidly Accelerated Fibrosarcoma, ROR: (adjusted) reporting odd-ratio, SLAMF7: signal lymphocytic activation molecule family member 7 and VEGF: vascular endothelial growth factor. Adjustment variables: age, sex, region, concurrent diseases or conditions of interest: hypertension, pulmonary embolism and/or deep venous thrombosis, acute coronary syndrome, supraventricular or ventricular tachyarrhythmias, torsade de pointes or QT prolongation, cardiac conduction disorders, cardiogenic shock, hyperthyroidism, cardiac valve disorders and myocarditis and all anticancer drug intakes, see main document and Supplementary Data 1. Yellow : analysis where the lower boundary of the CI is ≥1 ; orange, ≥2 ; dark orange, ≥5 and red, ≥ 10.

				Primary analysis		Sensitivity analyses											
						Primary analysis population				L-class pop.		Full database pop.		No HF medication pop.			
						multivariate		univariate		univariate		univariate		univariate			
Drug	Year 1st report	HF cases	Total reports	ROR	95%CI	ROR	95%CI	ROR	95%CI	ROR	95%CI	ROR	95%CI	ROR	95%CI	IC025	N signif.
Alkylating agents																	
Aziridines																	
- altretamine	1982	2	113	-		-		-		-		-		-		-	-
- mitomycin	1974	113	5438	-	-	2.08	(1.67 - 2.61)	2.02	(1.67-2.43)	2.04	(1.69-2.46)	3.65	(3.03-4.40)	2.34	(1.93-2.85)	0.71	6
- thiotepa	1968	68	4204	-	-	1.04	(0.72 - 1.50)	1.56	(1.23-1.99)	1.58	(1.24-2.01)	2.83	(2.23-3.60)	1.73	(1.35-2.23)	0.27	5
Nitrosoureas																	
- carmustine	1971	78	4080	-	-	2.16	(1.63 - 2.86)	1.85	(1.48-2.32)	1.87	(1.50-2.34)	3.35	(2.68-4.20)	2.12	(1.68-2.68)	0.53	6
- estramustine	1975	74	1811	-	-	3.10	(2.34 - 4.11)	4.05	(3.21-5.11)	4.10	(3.25-5.17)	7.33	(5.81-9.25)	4.18	(3.17-5.51)	1.60	6
- fotemustine	1990	2	480	-		-		-		-		-		-		-	-
- lomustine	1976	14	2566	-	-	0.69	(0.37 - 1.28)	0.52	(0.31-0.88)	0.53	(0.31-0.89)	0.94	(0.56-1.60)	0.62	(0.36-1.07)	-1.76	0
- streptozocin	1978	7	456	-	-	1.97	(0.87 - 4.47)	1.48	(0.70-3.12)	1.50	(0.71-3.16)	2.68	(1.27-5.66)	1.83	(0.87-3.85)	-0.75	1
Nitrogen mustards																	
- bendamustine	1996	211	16610	-	-	0.97	(0.81 - 1.16)	1.22	(1.07-1.40)	1.24	(1.08-1.42)	2.22	(1.93-2.54)	1.30	(1.12-1.51)	0.08	5
- chlorambucil	1968	36	4024	-	-	0.55	(0.37 - 0.82)	0.86	(0.62-1.19)	0.87	(0.62-1.20)	1.55	(1.12-2.16)	1.00	(0.69-1.43)	-0.73	1
- chlormethine	1969	20	3157	-	-	0.61	(0.37 - 1.02)	0.61	(0.39-0.94)	0.61	(0.39-0.95)	1.10	(0.71-1.70)	0.53	(0.31-0.91)	-1.41	0
- cyclophosphamide	1968	3157	196744	-	-	0.96	(0.90 - 1.02)	1.59	(1.54-1.65)	1.61	(1.55-1.67)	2.83	(2.74-2.94)	1.74	(1.68-1.81)	0.57	5
- ifosfamide	1974	206	23268	-	-	1.03	(0.87 - 1.21)	0.85	(0.74-0.97)	0.86	(0.75-0.98)	1.54	(1.34-1.76)	0.96	(0.84-1.11)	-0.44	1
- melphalan	1968	464	17585	-	-	1.13	(0.99 - 1.29)	2.59	(2.36-2.84)	2.62	(2.39-2.87)	4.67	(4.26-5.12)	2.60	(2.34-2.89)	1.20	5
Alkyl sulfonates																	
- busulfan	1968	160	9506	-	-	1.63	(1.32 - 2.02)	1.63	(1.39-1.90)	1.65	(1.41-1.93)	2.95	(2.52-3.45)	1.76	(1.49-2.08)	0.46	6
Platinum based drugs																	
- carboplatin	1986	937	151935	0.56	(0.52-0.61)	0.52	(0.47 - 0.56)	0.58	(0.54-0.62)	0.59	(0.55-0.63)	1.07	(1.00-1.14)	0.60	(0.56-0.65)	-0.85	1
- cisplatin	1977	845	154847	0.59	(0.54-0.64)	0.58	(0.53 - 0.63)	0.51	(0.48-0.55)	0.52	(0.48-0.55)	0.94	(0.88-1.01)	0.53	(0.50-0.57)	-1.03	0
- oxaliplatin	1997	477	149383	0.45	(0.41-0.5)	0.41	(0.36 - 0.45)	0.30	(0.27-0.32)	0.30	(0.27-0.33)	0.55	(0.50-0.60)	0.29	(0.26-0.32)	-1.84	0
Monofunctional alkylating agents																	
- dacarbazine	1976	116	8444	-	-	0.64	(0.48 - 0.86)	1.32	(1.10-1.59)	1.34	(1.11-1.61)	2.40	(2.00-2.88)	1.45	(1.19-1.76)	0.12	5
- temozolomide	1997	105	22345	-	-	0.54	(0.42 - 0.68)	0.45	(0.37-0.54)	0.45	(0.37-0.55)	0.81	(0.67-0.98)	0.46	(0.37-0.57)	-1.43	0
- procarbazine	1969	43	6461	-	-	1.04	(0.72 - 1.50)	0.64	(0.47-0.86)	0.64	(0.48-0.87)	1.15	(0.85-1.56)	0.60	(0.42-0.85)	-1.10	0
Tetrahydro-isoquinolines																	
- trabectedin	2004	99	2559	4.12	(3.27-5.18)	3.76	(2.98 - 4.74)	3.83	(3.13-4.68)	3.87	(3.17-4.73)	6.93	(5.67-8.47)	3.72	(2.95-4.68)	1.57	7
- lurbinectedin	2013	1	415	-		-		-		-		-		-		-	-
Others alkylating agents																	
- pipobroman	1985	0	225	-		-		-		-		-		-		-	-
ADT																	
Androgen receptor inhibitors																	
- apalutamide	2016	54	8104	-	-	0.64	(0.45 - 0.89)	0.64	(0.49-0.83)	0.64	(0.49-0.84)	1.15	(0.88-1.51)	0.61	(0.44-0.83)	-1.05	0
- bicalutamide	1995	364	15464	1.31	(1.16-1.48)	1.53	(1.35 - 1.74)	2.30	(2.07-2.55)	2.33	(2.09-2.58)	4.15	(3.74-4.61)	1.91	(1.64-2.22)	1.02	7
- cyproterone	1972	38	5348	-	-	0.78	(0.55 - 1.12)	0.68	(0.49-0.93)	0.93	(0.57-1.52)	1.23	(0.90-1.69)	0.54	(0.35-0.83)	-1.04	0
- darolutamide	2014	12	1250	-	-	0.94	(0.46 - 1.89)	0.92	(0.52-1.63)	0.93	(0.53-1.64)	1.67	(0.94-2.95)	0.84	(0.42-1.68)	-1.04	0
- enzalutamide	2012	491	51160	-	-	0.84	(0.75 - 0.94)	0.92	(0.84-1.01)	0.93	(0.85-1.02)	1.67	(1.53-1.82)	0.84	(0.76-0.94)	-0.25	1
- flutamide	1985	63	4812	-	-	0.99	(0.74 - 1.33)	1.26	(0.98-1.62)	1.27	(0.99-1.63)	2.28	(1.78-2.93)	1.19	(0.88-1.62)	-0.05	1
- nilutamide	1988	12	603	-	-	1.11	(0.53 - 2.34)	1.93	(1.09-3.42)	1.95	(1.10-3.45)	3.49	(1.97-6.19)	2.47	(1.32-4.63)	-0.05	4

				Primary analysis		Sensitivity analyses											
						Primary analysis population				L-class pop.		Full database pop.		No HF medication pop.			
						multivariate		univariate		univariate		univariate		univariate			
Drug	Year 1st report	HF cases	Total reports	ROR	95%CI	ROR	95%CI	ROR	95%CI	ROR	95%CI	ROR	95%CI	ROR	95%CI	IC025	N signif.
CYP-17 inhibitors																	
- abiraterone	2008	578	39251	1.48	(1.34-1.64)	1.35	(1.22 - 1.50)	1.43	(1.31-1.55)	1.44	(1.33-1.56)	2.58	(2.37-2.80)	1.44	(1.31-1.58)	0.38	7
GnRH agonists																	
- buserelin	1986	9	1356	-	-	0.62	(0.27 - 1.40)	0.63	(0.33-1.22)	0.64	(0.33-1.24)	1.15	(0.60-2.21)	0.55	(0.25-1.22)	-1.72	0
- goserelin	1986	218	15662	-	-	1.19	(1.02 - 1.39)	1.34	(1.17-1.54)	1.36	(1.19-1.55)	2.43	(2.13-2.78)	1.28	(1.09-1.51)	0.22	6
- histrelin	1995	3	947	-		-		-		-		-		-		-	-
- leuprorelin	1978	635	67174	-	-	0.87	(0.79 - 0.97)	0.91	(0.84-0.98)	0.92	(0.85-0.99)	1.64	(1.52-1.78)	0.71	(0.64-0.79)	-0.25	1
- triptorelin	1991	75	9290	-	-	1.02	(0.79 - 1.31)	0.77	(0.62-0.97)	0.78	(0.62-0.98)	1.40	(1.12-1.76)	0.61	(0.46-0.83)	-0.71	1
GnRH antagonists																	
- degarelix	2006	73	6111	-	-	1.13	(0.87 - 1.47)	1.15	(0.91-1.45)	1.16	(0.92-1.46)	2.08	(1.65-2.62)	1.04	(0.77-1.39)	-0.15	1
- relugolix	2017	14	4631	-	-	0.32	(0.16 - 0.64)	0.29	(0.17-0.49)	0.29	(0.17-0.49)	0.52	(0.31-0.88)	0.18	(0.08-0.39)	-2.60	0
Anthracyclines																	
- daunorubicin	1970	500	14663	2.99	(2.66-3.36)	3.46	(3.02 - 3.97)	3.38	(3.09-3.70)	3.42	(3.12-3.74)	6.09	(5.57-6.66)	3.85	(3.50-4.22)	1.58	7
- doxorubicin	1974	2916	133357	2.53	(2.41-2.66)	2.90	(2.72 - 3.09)	2.21	(2.12-2.29)	2.22	(2.14-2.31)	3.89	(3.75-4.03)	2.47	(2.37-2.57)	1.02	7
- epirubicin	1985	749	35205	2.71	(2.49-2.96)	3.01	(2.73 - 3.31)	2.08	(1.94-2.24)	2.11	(1.96-2.26)	3.75	(3.49-4.03)	2.34	(2.17-2.53)	0.92	7
- idarubicin	1987	175	8630	-	-	1.89	(1.57 - 2.28)	1.97	(1.70-2.29)	1.99	(1.71-2.31)	3.56	(3.07-4.14)	2.13	(1.81-2.51)	0.74	6
- mitoxantrone	1984	529	7867	5.05	(4.5-5.67)	6.12	(5.42 - 6.91)	6.92	(6.33-7.56)	6.99	(6.40-7.64)	12.43	(11.38-13.58)	7.80	(7.10-8.57)	2.56	7
Anti-aromatase																	
- anastrozole	1996	401	34095	-	-	0.81	(0.72 - 0.92)	1.13	(1.03-1.25)	1.14	(1.04-1.26)	2.05	(1.86-2.26)	0.93	(0.81-1.06)	0.03	4
- exemestane	2000	356	21889	-	-	1.17	(1.02 - 1.34)	1.58	(1.42-1.75)	1.59	(1.43-1.77)	2.85	(2.56-3.16)	1.39	(1.21-1.59)	0.49	6
- letrozole	1997	625	59215	-	-	1.01	(0.92 - 1.12)	1.01	(0.94-1.10)	1.02	(0.95-1.11)	1.84	(1.70-1.99)	0.93	(0.84-1.03)	-0.10	1
Antimetabolites																	
Antipyrimidiques																	
- azacitidine	1978	352	19840	-	-	1.58	(1.39 - 1.80)	1.72	(1.55-1.91)	1.74	(1.57-1.93)	3.11	(2.80-3.46)	1.58	(1.38-1.80)	0.61	6
- capecitabine	1999	905	103021	-	-	0.79	(0.73 - 0.86)	0.84	(0.78-0.90)	0.85	(0.79-0.91)	1.53	(1.43-1.63)	0.89	(0.83-0.96)	-0.34	1
- cytarabine	1970	1009	60400	-	-	1.11	(1.00 - 1.23)	1.63	(1.53-1.73)	1.65	(1.55-1.75)	2.93	(2.76-3.12)	1.75	(1.63-1.87)	0.59	6
- decitabine	2003	94	6900	-	-	1.11	(0.87 - 1.42)	1.31	(1.07-1.61)	1.33	(1.08-1.63)	2.38	(1.94-2.91)	1.20	(0.93-1.53)	0.08	4
- floxuridine	1975	1	333	-		-		-		-		-		-		-	-
- fluorouracil	1969	1157	141176	-	-	1.14	(1.06 - 1.23)	0.78	(0.73-0.83)	0.79	(0.74-0.84)	1.42	(1.34-1.51)	0.83	(0.78-0.88)	-0.43	2
- gemcitabine	1994	1032	94322	-	-	1.66	(1.54 - 1.79)	1.05	(0.99-1.12)	1.06	(1.00-1.13)	1.91	(1.79-2.03)	1.07	(0.99-1.14)	-0.02	2
- gimeracil	2008	0	14	-		-		-		-		-		-		-	-
- oteracil	2008	0	17	-		-		-		-		-		-		-	-
- tegafur	1976	1	981	-		-		-		-		-		-		-	-
Antipuriques																	
- cladribine	1993	36	17692	0.33	(0.22-0.5)	0.27	(0.18 - 0.41)	0.19	(0.14-0.27)	0.20	(0.14-0.27)	0.35	(0.25-0.49)	0.20	(0.14-0.29)	-2.85	0
- clofarabine	2005	121	3405	2.62	(2.07-3.32)	2.61	(2.05 - 3.32)	3.51	(2.92-4.21)	3.54	(2.96-4.25)	6.34	(5.29-7.61)	3.20	(2.58-3.96)	1.49	7
- fludarabine	1991	406	22897	1.63	(1.45-1.84)	1.33	(1.16 - 1.53)	1.72	(1.56-1.90)	1.74	(1.58-1.92)	3.11	(2.82-3.43)	1.80	(1.61-2.00)	0.62	7
- mercaptopurine	1969	118	21210	-	-	0.71	(0.57 - 0.88)	0.53	(0.44-0.64)	0.54	(0.45-0.64)	0.96	(0.80-1.15)	0.54	(0.44-0.66)	-1.17	0
- nelarabine	2006	2	565	-		-		-		-		-		-		-	-
- pentostatin	1983	43	951	-	-	3.56	(2.29 - 5.55)	4.50	(3.31-6.11)	4.55	(3.35-6.18)	8.15	(6.00-11.07)	4.15	(2.90-5.94)	1.60	6
- tioguanine	1971	33	3375	-	-	0.70	(0.45 - 1.10)	0.94	(0.67-1.32)	0.95	(0.67-1.34)	1.70	(1.21-2.39)	1.12	(0.79-1.59)	-0.62	1
Thymidine phosphorylase inhibitors																	
- tipiracil;trifluridine	2013	53	10130	-	-	0.54	(0.40 - 0.74)	0.50	(0.38-0.65)	0.50	(0.39-0.66)	0.91	(0.69-1.19)	0.43	(0.30-0.62)	-1.40	0
Folate analog metabolic inhibitor																	
- methotrexate	1969	2926	423422	0.72	(0.69-0.76)	0.78	(0.74 - 0.82)	0.64	(0.61-0.66)	0.65	(0.62-0.67)	1.20	(1.16-1.24)	0.56	(0.54-0.59)	-0.64	1
- pemetrexed	2000	256	34931	-	-	1.03	(0.89 - 1.20)	0.70	(0.62-0.79)	0.71	(0.63-0.80)	1.27	(1.12-1.44)	0.69	(0.60-0.80)	-0.69	1
- pralatrexate	2010	4	466	-		-		-		-		-		-		-	-
- raltitrexed	1997	11	2357	-	-	1.02	(0.56 - 1.84)	0.45	(0.25-0.81)	0.45	(0.25-0.81)	0.81	(0.45-1.46)	0.54	(0.30-0.98)	-2.10	0
Other antimetabolites																	
- hydroxycarbamide	1971	382	24398	-	-	0.97	(0.85 - 1.10)	1.52	(1.37-1.68)	1.53	(1.38-1.70)	2.74	(2.48-3.03)	1.32	(1.15-1.52)	0.44	5
Autologous cellular immunotherapy																	
- sipuleucel-t	2006	78	5025	-	-	0.96	(0.71 - 1.29)	1.50	(1.20-1.87)	2.37	(1.63-3.44)	2.71	(2.17-3.39)	0.93	(0.66-1.31)	0.23	4
Bcl-2 inhibitor																	
- venetoclax	2011	368	34839	-	-	0.82	(0.71 - 0.95)	1.01	(0.91-1.12)	1.03	(0.92-1.14)	1.84	(1.66-2.04)	0.84	(0.74-0.96)	-0.13	1
CAR-T cells																	
- axicabtagene ciloleucel	2018	63	5385	-	-	1.51	(1.14 - 2.00)	1.12	(0.88-1.44)	1.14	(0.89-1.46)	2.04	(1.59-2.61)	1.19	(0.91-1.56)	-0.21	2
- brexucabtagene autoleucel	2018	7	943	-	-	0.36	(0.11 - 1.14)	0.71	(0.34-1.49)	0.72	(0.34-1.51)	1.29	(0.61-2.71)	0.78	(0.35-1.73)	-1.72	0
- lisocabtagene maraleucel	2018	2	269	-		-		-		-		-		-		-	-
- tisagenlecleucel	2015	58	3339	-	-	2.45	(1.83 - 3.27)	1.68	(1.30-2.18)	1.70	(1.31-2.20)	3.04	(2.35-3.94)	1.84	(1.40-2.42)	0.33	6
Cytotoxic agents (not otherwise classified)																	
Asparagine-specific enzymes																	
- asparaginase	1970	83	13688	-	-	0.51	(0.39 - 0.67)	0.58	(0.47-0.72)	0.59	(0.47-0.73)	1.05	(0.85-1.30)	0.62	(0.49-0.77)	-1.10	0
- calaspargase pegol	2016	0	133	-		-		-		-		-		-		-	-

Drug	Year 1st report	HF cases	Total reports	Primary analysis		Sensitivity analyses: Primary analysis population, multivariate		Primary analysis population, univariate		L-class pop. univariate		Full database pop. univariate		No HF medication pop. univariate		IC025	N signif.
				ROR	95%CI	ROR	95%CI	ROR	95%CI	ROR	95%CI	ROR	95%CI	ROR	95%CI		
- crisantaspase	1994	3	547	-		-		-		-		-		-		-	-
- pegaspargase	1996	85	10891	-	-	0.61	(0.45 - 0.81)	0.75	(0.60-0.92)	0.75	(0.61-0.93)	1.35	(1.09-1.68)	0.90	(0.73-1.12)	-0.74	1
Modified oncolytic viral therapy																	
- talimogene laherparepvec	2009	9	1253	-	-	0.99	(0.46 - 2.10)	0.69	(0.36-1.32)	0.69	(0.36-1.34)	1.25	(0.65-2.40)	0.76	(0.38-1.53)	-1.61	0
Retinoid receptor agonists																	
- alitretinoin	2000	7	1283	-	-	1.09	(0.49 - 2.44)	0.52	(0.25-1.10)	0.53	(0.25-1.11)	0.94	(0.45-1.98)	0.48	(0.20-1.14)	-2.15	0
- bexarotene	2000	19	1315	-	-	1.45	(0.87 - 2.41)	1.39	(0.89-2.19)	1.41	(0.90-2.21)	2.52	(1.60-3.97)	1.21	(0.69-2.14)	-0.26	1
- tretinoin	1973	106	12064	-	-	0.92	(0.73 - 1.16)	0.84	(0.69-1.02)	0.85	(0.70-1.03)	1.53	(1.26-1.85)	0.78	(0.62-0.98)	-0.53	1
Other cytotoxic agents																	
- arsenic trioxide	1972	61	3083	-	-	1.81	(1.32 - 2.49)	1.92	(1.49-2.47)	1.94	(1.51-2.50)	3.47	(2.70-4.48)	1.70	(1.26-2.30)	0.53	6
- bleomycin	1971	136	13177	-	-	1.03	(0.81 - 1.31)	0.99	(0.84-1.17)	1.00	(0.85-1.19)	1.80	(1.52-2.13)	1.12	(0.94-1.33)	-0.27	1
- tasonermin	1999	1	180	-		-		-		-		-		-		-	-
Estrogen Agonists/Antagonists																	
- elacestrant	2020	7	1597	-	-	0.48	(0.23 - 1.01)	0.42	(0.20-0.88)	0.42	(0.20-0.89)	0.76	(0.36-1.59)	0.52	(0.23-1.16)	-2.45	0
- fulvestrant	1999	256	26324	-	-	1.01	(0.86 - 1.18)	0.93	(0.82-1.05)	0.94	(0.83-1.07)	1.69	(1.49-1.91)	0.85	(0.73-0.99)	-0.28	1
- medroxyprogesterone	1969	192	67155	0.27	(0.22-0.32)	0.40	(0.33 - 0.48)	0.27	(0.23-0.31)	0.27	(0.24-0.31)	0.49	(0.43-0.57)	0.18	(0.15-0.22)	-2.07	0
- megestrol	1971	389	22239	-	-	0.97	(0.85 - 1.10)	1.70	(1.53-1.88)	1.72	(1.55-1.90)	3.07	(2.77-3.39)	0.78	(0.64-0.96)	0.60	4
- tamoxifen	1976	443	41499	-	-	0.86	(0.77 - 0.96)	1.03	(0.93-1.13)	1.04	(0.94-1.14)	1.86	(1.69-2.04)	0.93	(0.83-1.05)	-0.10	1
- toremifene	1987	5	534	-	-	0.64	(0.26 - 1.62)	0.90	(0.37-2.17)	0.91	(0.38-2.19)	1.63	(0.67-3.92)	1.27	(0.53-3.06)	-1.67	0
FLT3 inhibitor																	
- midostaurin	2004	60	2802	-	-	1.84	(1.36 - 2.50)	2.08	(1.61-2.69)	2.10	(1.63-2.72)	3.77	(2.92-4.86)	2.07	(1.55-2.77)	0.64	6
Hedgehog pathway inhibitors																	
- glasdegib	2012	2	310	-		-		-		-		-		-		-	-
- sonidegib	2013	10	1656	-	-	0.49	(0.25 - 0.94)	0.58	(0.31-1.07)	0.58	(0.31-1.09)	1.05	(0.56-1.95)	0.29	(0.09-0.91)	-1.79	0
- vismodegib	2011	38	7917	-	-	0.53	(0.35 - 0.82)	0.46	(0.33-0.63)	0.46	(0.34-0.64)	0.83	(0.60-1.14)	0.35	(0.23-0.54)	-1.60	0
Histone deacetylase inhibitors																	
- belinostat	2008	5	248	-	-	1.19	(0.37 - 3.83)	1.95	(0.81-4.74)	1.98	(0.82-4.79)	3.54	(1.46-8.58)	1.01	(0.25-4.08)	-0.69	1
- panobinostat	2009	23	2035	-	-	0.57	(0.33 - 0.98)	1.09	(0.72-1.64)	1.10	(0.73-1.66)	1.97	(1.30-2.97)	1.26	(0.81-1.95)	-0.53	1
- romidepsin	2009	16	1011	-	-	1.51	(0.84 - 2.72)	1.53	(0.93-2.50)	1.54	(0.94-2.53)	2.77	(1.69-4.54)	1.12	(0.58-2.16)	-0.21	1
- vorinostat	2006	29	1840	-	-	0.98	(0.65 - 1.49)	1.52	(1.05-2.20)	1.54	(1.07-2.22)	2.76	(1.91-3.98)	1.50	(0.98-2.31)	0.01	4
Immunomodulating agents																	
- aldesleukin	1991	64	1856	-	-	2.60	(1.92 - 3.53)	3.40	(2.65-4.36)	4.56	(3.05-6.81)	6.15	(4.79-7.89)	3.90	(3.00-5.08)	1.33	6
- lenalidomide	2004	3978	336626	-	-	0.89	(0.85 - 0.94)	1.15	(1.11-1.19)	1.16	(1.12-1.20)	2.08	(2.01-2.14)	1.09	(1.05-1.14)	0.14	5
- pomalidomide	2010	680	73339	-	-	0.72	(0.65 - 0.79)	0.89	(0.82-0.96)	0.90	(0.83-0.97)	1.61	(1.50-1.74)	0.87	(0.79-0.95)	-0.28	1
- thalidomide	1969	918	42908	1.34	(1.24-1.45)	1.68	(1.55 - 1.83)	2.10	(1.97-2.24)	2.12	(1.99-2.27)	3.77	(3.53-4.03)	2.02	(1.87-2.18)	0.94	7
Immunotherapies																	
Immune checkpoint inhibitors																	
CTLA4 directed blocking antibody																	
- ipilimumab	2006	196	31877	-	-	0.61	(0.50 - 0.73)	0.59	(0.51-0.67)	0.59	(0.51-0.68)	1.06	(0.93-1.23)	0.61	(0.52-0.71)	-0.97	0
- tremelimumab	2010	4	591	-		-		-		-		-		-		-	-
Programmed Death Receptor-1 blocking antibody																	
- cemiplimab	2018	26	2014	-	-	1.01	(0.60 - 1.70)	1.24	(0.84-1.83)	1.26	(0.85-1.85)	2.25	(1.53-3.31)	1.14	(0.71-1.84)	-0.31	1
- dostarlimab	2019	3	379	-		-		-		-		-		-		-	-
- nivolumab	2012	612	76122	-	-	1.02	(0.91 - 1.13)	0.77	(0.71-0.83)	0.78	(0.72-0.84)	1.40	(1.29-1.51)	0.76	(0.69-0.83)	-0.49	1
- pembrolizumab	2006	552	59194	-	-	1.00	(0.90 - 1.11)	0.89	(0.82-0.97)	0.90	(0.83-0.98)	1.62	(1.49-1.76)	0.94	(0.85-1.03)	-0.28	1
- retifanlimab	2019	0	28	-		-		-		-		-		-		-	-
- toripalimab	2021	0	38	-		-		-		-		-		-		-	-
Programmed Death Ligand-1 blockers																	
- atezolizumab	2012	172	17800	-	-	0.80	(0.67 - 0.95)	0.93	(0.80-1.08)	0.94	(0.81-1.09)	1.68	(1.45-1.95)	0.93	(0.78-1.12)	-0.33	1
- avelumab	2015	28	4357	-	-	0.45	(0.27 - 0.74)	0.61	(0.42-0.89)	0.62	(0.43-0.90)	1.11	(0.77-1.61)	0.60	(0.39-0.93)	-1.27	0
- durvalumab	2014	54	7864	-	-	0.76	(0.54 - 1.08)	0.66	(0.50-0.86)	0.66	(0.51-0.87)	1.19	(0.91-1.56)	0.70	(0.52-0.94)	-1.01	0
Interferon																	
- interferon alfa-2b	1987	141	17249	-	-	1.13	(0.93 - 1.38)	0.78	(0.66-0.92)	2.06	(1.39-3.03)	1.42	(1.20-1.67)	0.81	(0.67-0.97)	-0.60	2
Kinase inhibitors																	
AKT inhibitors																	
- capivasertib	2020	0	31	-		-		-		-		-		-		-	-
ALK inhibitors																	
- alectinib	2014	79	6437	-	-	1.68	(1.26 - 2.24)	1.18	(0.95-1.47)	1.19	(0.96-1.49)	2.14	(1.71-2.67)	1.18	(0.92-1.52)	-0.10	2
- brigatinib	2016	15	2295	-	-	1.14	(0.64 - 2.05)	0.62	(0.38-1.04)	0.63	(0.38-1.05)	1.13	(0.68-1.88)	0.54	(0.29-1.01)	-1.48	0
- ceritinib	2014	19	3315	-	-	0.64	(0.34 - 1.20)	0.55	(0.35-0.86)	0.55	(0.35-0.87)	0.99	(0.63-1.56)	0.53	(0.32-0.88)	-1.57	0
- crizotinib	2010	181	13375	-	-	1.83	(1.55 - 2.16)	1.30	(1.13-1.51)	1.32	(1.14-1.53)	2.36	(2.04-2.74)	1.34	(1.14-1.58)	0.16	6
- lorlatinib	2016	47	3817	-	-	1.86	(1.34 - 2.56)	1.18	(0.89-1.58)	1.20	(0.90-1.60)	2.15	(1.61-2.86)	1.13	(0.82-1.57)	-0.20	2

Drug	Year 1st report	HF cases	Total reports	Primary analysis ROR	Primary analysis 95%CI	Sensitivity analyses: Primary analysis population, multivariate ROR	95%CI	Primary analysis population, univariate ROR	95%CI	L-class pop. univariate ROR	95%CI	Full database pop. univariate ROR	95%CI	No HF medication pop. univariate ROR	95%CI	IC025	N signif.
BCR-ABL inhibitors																	
- asciminib	2015	11	958	-	-	1.07	(0.50 - 2.27)	1.10	(0.61-2.00)	1.12	(0.62-2.02)	2.00	(1.10-3.62)	0.80	(0.36-1.78)	-0.84	1
- bosutinib	2009	233	7525	2.75	(2.35-3.22)	2.36	(2.02 - 2.77)	3.05	(2.67-3.47)	3.08	(2.70-3.51)	5.50	(4.83-6.27)	2.94	(2.52-3.43)	1.37	7
- dasatinib	2003	1415	35065	5.03	(4.7-5.39)	4.75	(4.42 - 5.10)	4.10	(3.88-4.32)	4.13	(3.92-4.36)	7.28	(6.90-7.68)	4.20	(3.95-4.46)	1.88	7
- imatinib	2002	1116	69004	1.74	(1.61-1.88)	1.83	(1.69 - 1.98)	1.58	(1.48-1.67)	1.59	(1.50-1.69)	2.84	(2.68-3.01)	1.54	(1.43-1.65)	0.55	7
- nilotinib	2006	510	32097	-	-	1.32	(1.16 - 1.49)	1.54	(1.41-1.68)	1.56	(1.43-1.70)	2.78	(2.55-3.04)	1.56	(1.41-1.72)	0.48	6
- ponatinib	2011	214	8780	1.91	(1.59-2.3)	1.75	(1.45 - 2.10)	2.38	(2.08-2.73)	2.41	(2.10-2.76)	4.30	(3.76-4.93)	2.28	(1.94-2.68)	1.02	7
BTK inhibitors																	
- acalabrutinib	2016	55	5305	-	-	0.87	(0.60 - 1.27)	1.00	(0.76-1.30)	1.01	(0.77-1.31)	1.80	(1.38-2.35)	1.06	(0.79-1.43)	-0.41	1
- ibrutinib	2013	1312	68915	1.52	(1.41-1.64)	1.34	(1.24 - 1.45)	1.87	(1.77-1.98)	1.89	(1.79-2.00)	3.35	(3.18-3.54)	1.68	(1.57-1.80)	0.79	7
- pirtobrutinib	2021	0	105	-		-		-		-		-		-		-	-
- zanubrutinib	2015	15	1213	-	-	0.97	(0.43 - 2.21)	1.19	(0.71-1.98)	1.20	(0.72-2.00)	2.15	(1.29-3.59)	1.18	(0.65-2.14)	-0.58	1
CDK inhibitors																	
- abemaciclib	2014	77	15974	-	-	0.78	(0.60 - 1.02)	0.46	(0.37-0.57)	0.46	(0.37-0.58)	0.83	(0.67-1.04)	0.47	(0.37-0.60)	-1.45	0
- palbociclib	2012	566	92513	-	-	0.74	(0.68 - 0.82)	0.58	(0.53-0.63)	0.59	(0.54-0.64)	1.06	(0.98-1.15)	0.63	(0.58-0.69)	-0.89	0
- ribociclib	2014	166	22319	-	-	1.20	(0.98 - 1.46)	0.71	(0.61-0.83)	0.72	(0.62-0.84)	1.29	(1.11-1.50)	0.73	(0.62-0.87)	-0.71	1
- trilaciclib	2021	1	104	-		-		-		-		-		-		-	-
Colony-stimulating factor-1 receptor (CSF-1R)																	
- pexidartinib	2016	0	689	-		-		-		-		-		-		-	-
EGFR inhibitors																	
- afatinib	2009	66	11983	-	-	0.70	(0.53 - 0.92)	0.53	(0.41-0.67)	0.53	(0.42-0.68)	0.95	(0.75-1.21)	0.56	(0.43-0.73)	-1.28	0
- dacomitinib	2012	4	897	-		-		-		-		-		-		-	-
- erlotinib	2003	351	43421	-	-	0.94	(0.81 - 1.08)	0.77	(0.69-0.86)	0.78	(0.70-0.87)	1.40	(1.26-1.56)	0.73	(0.64-0.83)	-0.52	1
- gefitinib	2002	86	10257	-	-	1.00	(0.79 - 1.27)	0.80	(0.65-0.99)	0.81	(0.66-1.00)	1.46	(1.18-1.80)	0.71	(0.55-0.92)	-0.63	1
- mobocertinib	2020	5	442	-	-	1.26	(0.46 - 3.41)	1.09	(0.45-2.62)	1.10	(0.46-2.65)	1.97	(0.82-4.75)	1.35	(0.50-3.61)	-1.42	0
- osimertinib	2014	436	19739	3.64	(3.24-4.09)	3.04	(2.70 - 3.42)	2.16	(1.96-2.37)	2.18	(1.98-2.40)	3.89	(3.54-4.28)	2.29	(2.07-2.55)	0.94	7
FGFR inhibitors																	
- erdafitinib	2018	3	1022	-		-		-		-		-		-		-	-
- futibatinib	2015	0	47	-		-		-		-		-		-		-	-
- infigratinib	2019	0	49	-		-		-		-		-		-		-	-
- pemigatinib	2020	1	569	-		-		-		-		-		-		-	-
FLT-3 inhibitors																	
- gilteritinib	2017	24	1160	-	-	2.12	(1.31 - 3.42)	2.01	(1.34-3.01)	2.03	(1.35-3.04)	3.64	(2.43-5.45)	2.46	(1.61-3.76)	0.33	6
- quizartinib	2015	0	30	-		-		-		-		-		-		-	-
JAK inhibitors																	
- fedratinib	2013	11	1033	-	-	1.19	(0.58 - 2.42)	1.02	(0.56-1.85)	1.03	(0.57-1.87)	1.85	(1.02-3.36)	0.79	(0.35-1.76)	-0.95	1
- momelotinib	2015	0	83	-		-		-		-		-		-		-	-
- pacritinib	2013	8	1226	-	-	0.80	(0.11 - 5.76)	0.62	(0.31-1.25)	0.63	(0.31-1.26)	1.13	(0.56-2.27)	0.69	(0.29-1.66)	-1.81	0
- ruxolitinib	2012	626	58126	-	-	1.32	(1.17 - 1.48)	1.03	(0.96-1.12)	1.05	(0.97-1.13)	1.88	(1.73-2.03)	0.80	(0.72-0.90)	-0.07	2
HER2 inhibitors																	
- lapatinib	2003	252	15846	-	-	0.88	(0.74 - 1.04)	1.54	(1.36-1.74)	1.55	(1.37-1.76)	2.78	(2.46-3.15)	1.72	(1.50-1.97)	0.43	5
- neratinib	2010	18	2351	-	-	1.47	(0.81 - 2.68)	0.73	(0.46-1.17)	0.74	(0.47-1.18)	1.33	(0.84-2.11)	0.77	(0.45-1.30)	-1.18	0
- tucatinib	2017	33	3158	-	-	0.55	(0.35 - 0.89)	1.00	(0.71-1.41)	1.01	(0.72-1.43)	1.82	(1.29-2.56)	0.99	(0.67-1.46)	-0.53	1
MEK inhibitors																	
- binimetinib	2014	47	4735	-	-	1.20	(0.64 - 2.26)	0.95	(0.71-1.27)	0.96	(0.72-1.28)	1.73	(1.29-2.30)	1.00	(0.72-1.40)	-0.51	1
- cobimetinib	2012	78	3075	-	-	2.85	(2.04 - 3.99)	2.47	(1.98-3.10)	2.50	(2.00-3.13)	4.48	(3.58-5.61)	2.50	(1.92-3.25)	0.93	6
- selumetinib	2010	19	986	-	-	3.24	(1.88 - 5.58)	1.87	(1.19-2.94)	1.89	(1.20-2.97)	3.38	(2.15-5.32)	1.97	(1.20-3.23)	0.13	6
- trametinib	2011	383	19923	3.47	(3.07-3.92)	2.26	(1.75 - 2.90)	1.87	(1.69-2.07)	1.89	(1.71-2.09)	3.38	(3.05-3.74)	1.97	(1.76-2.20)	0.73	7
MET inhibitors																	
- capmatinib	2014	21	1813	-	-	0.86	(0.35 - 2.11)	1.11	(0.72-1.71)	1.13	(0.73-1.73)	2.02	(1.31-3.10)	1.27	(0.81-1.99)	-0.53	1
- tepotinib	2019	7	473	-	-	1.54	(0.67 - 3.53)	1.43	(0.68-3.01)	1.44	(0.68-3.04)	2.59	(1.23-5.45)	0.56	(0.14-2.23)	-0.79	1
PI3K inhibitors																	
- alpelisib	2013	32	7572	-	-	0.71	(0.44 - 1.16)	0.40	(0.28-0.57)	0.41	(0.29-0.58)	0.73	(0.52-1.03)	0.38	(0.25-0.56)	-1.83	0
- copanlisib	2013	4	240	-		-		-		-		-		-		-	-
- duvelisib	2015	2	553	-		-		-		-		-		-		-	-
- idelalisib	2013	72	6839	-	-	1.13	(0.88 - 1.45)	1.01	(0.80-1.28)	1.02	(0.81-1.29)	1.83	(1.45-2.31)	0.92	(0.70-1.22)	-0.34	1
- umbralisib	2018	2	271	-		-		-		-		-		-		-	-
RAF inhibitors																	
- dabrafenib	2011	347	18325	-	-	1.49	(1.14 - 1.94)	1.84	(1.65-2.05)	1.86	(1.67-2.07)	3.33	(2.99-3.70)	1.92	(1.71-2.16)	0.70	6
- encorafenib	2014	52	6066	-	-	0.92	(0.51 - 1.65)	0.82	(0.62-1.08)	0.83	(0.63-1.09)	1.49	(1.13-1.96)	0.84	(0.61-1.16)	-0.70	1
- regorafenib	2009	91	16212	-	-	0.66	(0.52 - 0.83)	0.54	(0.44-0.66)	0.54	(0.44-0.67)	0.97	(0.79-1.19)	0.59	(0.46-0.74)	-1.20	0

Drug	Year 1st report	HF cases	Total reports	Primary analysis		Sensitivity analyses: Primary analysis population multivariate		Primary analysis population univariate		L-class pop. univariate		Full database pop. univariate		No HF medication pop. univariate		IC025	N signif.
				ROR	95%CI	ROR	95%CI	ROR	95%CI	ROR	95%CI	ROR	95%CI	ROR	95%CI		
RET inhibitors																	
- pralsetinib	2020	16	708	-	-	3.27	(1.83 - 5.84)	2.20	(1.34-3.61)	2.22	(1.35-3.65)	3.98	(2.42-6.53)	2.64	(1.58-4.41)	0.27	6
- selpercatinib	2019	12	907	-	-	1.60	(0.75 - 3.41)	1.27	(0.72-2.25)	1.29	(0.73-2.28)	2.31	(1.31-4.08)	1.26	(0.65-2.44)	-0.60	1
ROS-1 inhibitors																	
- repotrectinib	2022	0	12	-		-		-		-		-		-		-	-
TRK inhibitors																	
- entrectinib	2017	35	758	10.84	(7.24-16.24)	8.37	(5.58 - 12.55)	4.60	(3.28-6.46)	4.65	(3.31-6.53)	8.33	(5.93-11.70)	5.10	(3.54-7.35)	1.56	7
- larotrectinib	2019	3	651	-		-		-		-		-		-		-	-
VEGF inhibitors																	
- axitinib	2008	195	16048	-	-	1.15	(0.98 - 1.36)	1.17	(1.01-1.35)	1.18	(1.03-1.36)	2.12	(1.84-2.44)	1.24	(1.06-1.46)	0.01	5
- cabozantinib	2012	219	26290	-	-	0.92	(0.77 - 1.09)	0.80	(0.70-0.91)	0.81	(0.71-0.92)	1.45	(1.27-1.65)	0.83	(0.70-0.97)	-0.52	1
- fruquintinib	2020	0	271	-		-		-		-		-		-		-	-
- lenvatinib	2013	285	19204	-	-	1.60	(1.40 - 1.83)	1.43	(1.28-1.61)	1.45	(1.29-1.63)	2.59	(2.31-2.92)	1.56	(1.36-1.79)	0.34	6
- nintedanib	2008	371	27369	-	-	1.31	(1.16 - 1.47)	1.31	(1.18-1.45)	1.32	(1.19-1.47)	2.37	(2.14-2.62)	1.30	(1.16-1.47)	0.23	6
- pazopanib	2005	382	28536	-	-	1.64	(1.45 - 1.87)	1.29	(1.17-1.43)	1.31	(1.18-1.44)	2.34	(2.11-2.59)	1.37	(1.22-1.54)	0.21	6
- tivozanib	2010	14	1323	-		-		-		-		-		-		-	-
- sorafenib	2004	338	34601	-	-	0.98	(0.87 - 1.11)	0.94	(0.84-1.04)	0.95	(0.85-1.05)	1.70	(1.53-1.89)	0.89	(0.78-1.01)	-0.25	1
- sunitinib	2003	909	42181	1.97	(1.81-2.14)	1.98	(1.82 - 2.16)	2.12	(1.98-2.26)	2.14	(2.00-2.28)	3.80	(3.56-4.06)	2.23	(2.06-2.41)	0.95	7
- vandetanib	2006	10	1608	-	-	0.65	(0.31 - 1.39)	0.59	(0.32-1.11)	0.60	(0.32-1.12)	1.08	(0.58-2.01)	0.57	(0.27-1.20)	-1.75	0
- vemurafenib	2010	123	10503	-	-	1.12	(0.85 - 1.47)	1.13	(0.94-1.35)	1.14	(0.95-1.36)	2.04	(1.71-2.44)	1.11	(0.90-1.36)	-0.10	1
Others TKI																	
- avapritinib	2017	14	5116	-	-	0.35	(0.19 - 0.66)	0.26	(0.15-0.44)	0.26	(0.16-0.44)	0.47	(0.28-0.80)	0.23	(0.12-0.43)	-2.75	0
- ripretinib	2020	8	2661	-	-	0.62	(0.23 - 1.67)	0.29	(0.14-0.57)	0.29	(0.14-0.58)	0.52	(0.26-1.04)	0.23	(0.09-0.55)	-2.90	0
Mitotic spindle poisons																	
Halichondrin																	
- eribulin	2009	73	7975	-	-	0.97	(0.75 - 1.27)	0.88	(0.70-1.10)	0.89	(0.70-1.12)	1.59	(1.26-2.00)	0.84	(0.65-1.10)	-0.54	1
Microtubule inhibitors																	
- cabazitaxel	2009	33	5125	-	-	0.62	(0.42 - 0.92)	0.62	(0.44-0.87)	0.62	(0.44-0.88)	1.12	(0.79-1.57)	0.58	(0.39-0.86)	-1.22	0
- docetaxel	1995	1426	168577	0.55	(0.51-0.59)	0.55	(0.51 - 0.59)	0.80	(0.76-0.85)	0.81	(0.77-0.86)	1.47	(1.40-1.55)	0.82	(0.77-0.87)	-0.38	1
- ixabepilone	2004	27	1774	-	-	1.11	(0.69 - 1.79)	1.47	(1.00-2.15)	1.48	(1.01-2.17)	2.66	(1.82-3.89)	1.27	(0.80-2.02)	-0.06	3
- paclitaxel	1992	1886	200510	0.84	(0.79-0.89)	0.79	(0.74 - 0.84)	0.90	(0.86-0.94)	0.91	(0.87-0.95)	1.64	(1.57-1.72)	0.95	(0.90-1.00)	-0.21	1
Vinca alkaloids																	
- vinblastine	1969	145	8753	-	-	1.52	(1.18 - 1.97)	1.60	(1.36-1.89)	1.62	(1.37-1.91)	2.90	(2.46-3.42)	1.76	(1.48-2.09)	0.42	6
- vincristine	1968	1210	75369	-	-	0.92	(0.84 - 1.01)	1.57	(1.48-1.66)	1.58	(1.49-1.68)	2.82	(2.66-2.98)	1.69	(1.59-1.80)	0.54	5
- vindesine	1979	29	4754	-	-	0.45	(0.29 - 0.71)	0.58	(0.40-0.84)	0.59	(0.41-0.85)	1.06	(0.73-1.52)	0.68	(0.47-0.98)	-1.33	0
- vinflunine	2003	3	563	-		-		-		-		-		-		-	-
- vinorelbine	1990	348	18392	-	-	1.29	(1.13 - 1.47)	1.84	(1.65-2.05)	1.86	(1.67-2.07)	3.32	(2.99-3.69)	1.99	(1.77-2.23)	0.70	6
Monoclonal antibodies																	
anti-B-cell maturation antigen (BCMA)																	
- belantamab mafodotin	2020	12	1746	-	-	0.53	(0.23 - 1.26)	0.66	(0.37-1.16)	0.66	(0.38-1.17)	1.19	(0.68-2.10)	0.67	(0.36-1.24)	-1.51	0
- elranatamab	2021	3	188	-		-		-		-		-		-		-	-
- teclistamab	2021	7	1391	-	-	0.67	(0.28 - 1.63)	0.48	(0.23-1.01)	0.49	(0.23-1.02)	0.87	(0.41-1.83)	0.60	(0.28-1.26)	-2.26	0
Anti-CCR4																	
- mogamulizumab	2013	17	1827	-	-	1.04	(0.54 - 2.01)	0.89	(0.55-1.44)	0.90	(0.56-1.45)	1.62	(1.00-2.61)	0.97	(0.57-1.64)	-0.93	1
Anti-CD9																	
- blinatumomab	2013	36	6376	-	-	0.76	(0.51 - 1.12)	0.54	(0.39-0.75)	0.54	(0.39-0.76)	0.98	(0.70-1.36)	0.57	(0.40-0.81)	-1.38	0
Anti-CD19																	
- loncastuximab tesirine	2017	0	128	-		-		-		-		-		-		-	-
- tafasitamab	2015	10	965	-	-	1.06	(0.43 - 2.61)	0.99	(0.53-1.85)	1.01	(0.54-1.88)	1.80	(0.97-3.36)	0.85	(0.38-1.89)	-1.04	0
Anti-CD20																	
- epcoritamab	2020	3	493	-		-		-		-		-		-		-	-
- glofitamab	2018	3	400	-		-		-		-		-		-		-	-
- ibritumomab tiuxetan	2000	20	899	-	-	1.86	(1.08 - 3.20)	2.16	(1.39-3.37)	2.42	(1.42-4.11)	3.92	(2.51-6.10)	2.16	(1.32-3.55)	0.35	6
- mosunetuzumab	2019	2	239	-		-		-		-		-		-		-	-
- rituximab	1999	1653	144178	-	-	1.02	(0.96 - 1.10)	1.11	(1.05-1.16)	1.12	(1.06-1.17)	2.00	(1.91-2.10)	1.12	(1.06-1.19)	0.07	5
- obinutuzumab	2011	84	8175	-	-	1.04	(0.81 - 1.34)	0.99	(0.80-1.22)	1.00	(0.80-1.24)	1.79	(1.44-2.22)	0.88	(0.68-1.15)	-0.34	1
- ofatumumab	2008	30	24087	-	-	0.28	(0.18 - 0.45)	0.12	(0.08-0.17)	0.12	(0.08-0.17)	0.21	(0.15-0.31)	0.13	(0.09-0.19)	-3.61	0
Anti-CD22																	
- inotuzumab ozogamicin	2010	10	1762	-	-	0.61	(0.30 - 1.24)	0.54	(0.29-1.01)	0.55	(0.29-1.02)	0.98	(0.53-1.83)	0.56	(0.28-1.11)	-1.87	0
- moxetumomab pasudotox	2017	0	56	-		-		-		-		-		-		-	-
Anti-CD25																	
- basiliximab	1999	107	6453	-	-	2.06	(1.66 - 2.56)	1.60	(1.32-1.94)	1.04	(0.64-1.67)	2.90	(2.40-3.51)	1.73	(1.39-2.15)	0.38	5

Drug	Year 1st report	HF cases	Total reports	Primary analysis ROR	Primary analysis 95%CI	Sensitivity analyses: Primary analysis population, multivariate ROR	95%CI	Primary analysis population, univariate ROR	95%CI	L-class pop. univariate ROR	95%CI	Full database pop. univariate ROR	95%CI	No HF medication pop. univariate ROR	95%CI	IC025	N signif.
Anti-CD30																	
- brentuximab vedotin	2011	90	7933	-	-	1.36	(1.06 - 1.75)	1.09	(0.89-1.34)	1.10	(0.90-1.36)	1.98	(1.60-2.43)	1.14	(0.91-1.43)	-0.19	2
Anti-CD33																	
- gemtuzumab ozogamicin	2001	103	2712	1.77	(1.38-2.27)	1.67	(1.30 - 2.15)	3.76	(3.08-4.58)	3.80	(3.12-4.62)	6.80	(5.58-8.28)	4.00	(3.22-4.97)	1.56	7
Anti-CD38																	
- daratumumab	2010	338	22809	-	-	1.11	(0.97 - 1.26)	1.43	(1.29-1.60)	1.45	(1.30-1.61)	2.59	(2.33-2.89)	1.48	(1.31-1.68)	0.35	5
- isatuximab	2014	35	1642	-	-	0.93	(0.63 - 1.37)	2.07	(1.48-2.89)	2.09	(1.50-2.92)	3.75	(2.68-5.24)	2.19	(1.48-3.23)	0.49	5
Anti-CD52																	
- alemtuzumab	1995	195	24149	-	-	1.21	(1.02 - 1.44)	0.77	(0.67-0.89)	0.78	(0.68-0.90)	1.40	(1.22-1.61)	0.86	(0.74-1.00)	-0.58	2
Anti-CD123																	
- tagraxofusp	2019	3	320	-		-		-		-		-		-		-	-
Anti-GD2																	
- naxitamab	2018	1	176	-		-		-		-		-		-		-	-
Anti-gp100 peptide																	
- tebentafusp	2018	0	338	-		-		-		-		-		-		-	-
Anti-GPRC5D																	
- talquetamab	2022	0	213	-		-		-		-		-		-		-	-
Anti-LAG3																	
- relatlimab	2017	2	112	-		-		-		-		-		-		-	-
Anti-nectin-4																	
- enfortumab vedotin	2018	9	1670	-	-	0.76	(0.38 - 1.53)	0.51	(0.27-0.99)	0.52	(0.27-1.00)	0.93	(0.48-1.80)	0.23	(0.07-0.71)	-2.01	0
Anti-PDGFRa																	
- olaratumab	2010	11	603	-	-	0.83	(0.38 - 1.81)	1.77	(0.97-3.21)	1.78	(0.98-3.24)	3.20	(1.76-5.81)	1.81	(0.94-3.50)	-0.21	1
EGFR inhibitors																	
- cetuximab	2000	192	45611	0.61	(0.52-0.71)	0.63	(0.54 - 0.74)	0.40	(0.35-0.46)	0.40	(0.35-0.47)	0.73	(0.63-0.84)	0.39	(0.33-0.46)	-1.52	0
- necitumumab	2010	5	170	-	-	2.69	(0.95 - 7.61)	2.88	(1.18-7.01)	2.91	(1.20-7.08)	5.21	(2.14-12.70)	1.65	(0.41-6.68)	-0.25	3
- panitumumab	2004	63	15374	-	-	0.72	(0.55 - 0.95)	0.39	(0.30-0.50)	0.39	(0.31-0.51)	0.71	(0.55-0.91)	0.37	(0.28-0.50)	-1.72	0
EGFR-MET bispecific antibody																	
- amivantamab	2020	4	757	-		-		-		-		-		-		-	-
Folate receptor alpha																	
- mirvetuximab soravtansine	2017	1	238	-		-		-		-		-		-		-	-
Glycolipid disialoganglioside-directed antibody																	
- dinutuximab	2015	10	318	-	-	4.57	(2.09 - 9.99)	3.08	(1.64-5.79)	3.12	(1.66-5.85)	5.59	(2.98-10.49)	3.33	(1.72-6.46)	0.43	6
HER2 inhibitors																	
- margetuximab	2021	0	36	-		-		-		-		-		-		-	-
- pertuzumab	2006	857	19354	-	-	1.02	(0.92 - 1.13)	4.47	(4.17-4.79)	4.51	(4.21-4.84)	8.00	(7.47-8.57)	4.88	(4.52-5.26)	1.99	5
- trastuzumab	1999	4229	60951	11.65	(11.1-12.22)	11.81	(11.19 - 12.47)	7.75	(7.50-8.01)	7.78	(7.53-8.03)	13.07	(12.67-13.49)	9.31	(8.99-9.64)	2.69	7
- trastuzumab emtansine	2010	217	8022	1.68	(1.42-1.99)	1.52	(1.27 - 1.81)	2.65	(2.31-3.03)	2.68	(2.34-3.07)	4.79	(4.18-5.48)	2.79	(2.41-3.24)	1.17	7
- trastuzumab deruxtecan	2019	89	7006	-	-	2.07	(1.55 - 2.76)	1.22	(0.99-1.51)	1.24	(1.00-1.52)	2.21	(1.80-2.73)	1.29	(1.03-1.62)	-0.03	4
SLAMF7-directed immunostimulatory antibody																	
- elotuzumab	2010	51	3410	-	-	0.94	(0.66 - 1.33)	1.44	(1.09-1.90)	1.46	(1.11-1.92)	2.61	(1.98-3.45)	1.51	(1.08-2.09)	0.09	5
VEGF inhibitors																	
- aflibercept	2008	181	29711	-	-	1.00	(0.83 - 1.20)	0.58	(0.50-0.67)	0.59	(0.51-0.68)	1.05	(0.91-1.22)	0.51	(0.43-0.62)	-0.99	0
- bevacizumab	2000	1212	96520	-	-	1.85	(1.72 - 1.99)	1.21	(1.15-1.29)	1.23	(1.16-1.30)	2.20	(2.07-2.32)	1.30	(1.22-1.39)	0.19	6
- ramucirumab	2010	78	8917	-	-	1.55	(1.18 - 2.03)	0.84	(0.67-1.05)	0.85	(0.68-1.06)	1.52	(1.22-1.90)	0.80	(0.62-1.03)	-0.59	2
Others																	
- polatuzumab vedotin	2014	19	1891	-	-	1.01	(0.60 - 1.71)	0.96	(0.61-1.51)	0.97	(0.62-1.53)	1.75	(1.11-2.74)	1.06	(0.65-1.73)	-0.77	1
- sacituzumab govitecan	2020	11	3534	-	-	0.53	(0.24 - 1.20)	0.30	(0.16-0.54)	0.30	(0.17-0.54)	0.54	(0.30-0.97)	0.23	(0.11-0.49)	-2.67	0
- tisotumab vedotin	2016	0	298	-		-		-		-		-		-		-	-
mTOR inhibitors																	
- everolimus	2002	684	53092	-	-	1.48	(1.34 - 1.64)	1.24	(1.15-1.34)	1.26	(1.17-1.36)	2.25	(2.09-2.43)	1.18	(1.08-1.30)	0.20	6
- temsirolimus	2003	84	4053	-	-	1.61	(1.21 - 2.15)	2.01	(1.62-2.50)	2.03	(1.64-2.53)	3.64	(2.93-4.52)	1.87	(1.44-2.44)	0.66	6
Muramyl dipeptide derived agent																	
- mifamurtide	2010	0	216	-		-		-		-		-		-		-	-
Peptide receptor radionuclide therapy																	
- lutetium (177lu) dotatate	2014	13	3984	-	-	0.72	(0.33 - 1.54)	0.31	(0.18-0.54)	1.03	(0.26-4.16)	0.56	(0.33-0.97)	0.33	(0.18-0.60)	-2.53	0
- lutetium (177lu) vipivotide tetraxetan	2017	7	3274	-	-	0.35	(0.06 - 1.90)	0.20	(0.10-0.43)	0.61	(0.09-4.34)	0.37	(0.18-0.77)	0.18	(0.07-0.42)	-3.47	0
Poly(ADP-Ribose) Polymerase inhibitors																	
- niraparib	2015	102	18589	-	-	0.57	(0.42 - 0.78)	0.52	(0.43-0.64)	0.53	(0.44-0.64)	0.95	(0.78-1.15)	0.47	(0.37-0.60)	-1.22	0
- olaparib	2009	73	14371	-	-	0.77	(0.57 - 1.04)	0.48	(0.38-0.61)	0.49	(0.39-0.62)	0.88	(0.70-1.11)	0.49	(0.37-0.63)	-1.38	0
- rucaparib	2016	29	8152	-	-	0.58	(0.37 - 0.91)	0.34	(0.24-0.49)	0.34	(0.24-0.49)	0.61	(0.43-0.88)	0.28	(0.17-0.44)	-2.11	0
- talazoparib	2015	7	1543	-	-	0.70	(0.31 - 1.57)	0.43	(0.21-0.91)	0.44	(0.21-0.92)	0.78	(0.37-1.65)	0.47	(0.21-1.04)	-2.40	0

				Primary analysis		Sensitivity analyses											
						Primary analysis population				L-class pop.		Full database pop.		No HF medication pop.			
						multivariate		univariate		univariate		univariate		univariate			
Drug	Year 1st report	HF cases	Total reports	ROR	95%CI	ROR	95%CI	ROR	95%CI	ROR	95%CI	ROR	95%CI	ROR	95%CI	IC025	N signif.
Proteasom inhibitors																	
- bortezomib	2000	1396	65793	1.79	(1.67-1.92)	1.80	(1.68 - 1.94)	2.09	(1.98-2.21)	2.12	(2.00-2.23)	3.75	(3.55-3.95)	2.12	(1.99-2.26)	0.95	7
- carfilzomib	2009	1046	20700	5.21	(4.8-5.65)	5.03	(4.62 - 5.48)	5.16	(4.84-5.49)	5.21	(4.89-5.54)	9.20	(8.64-9.79)	5.84	(5.46-6.26)	2.19	7
- ixazomib	2013	194	18767	-	-	1.05	(0.88 - 1.26)	0.99	(0.86-1.14)	1.00	(0.87-1.16)	1.80	(1.56-2.07)	0.97	(0.81-1.15)	-0.22	1
Topoisomerase inhibitors																	
Type 1 topoisomerase inhibitors																	
- irinotecan	1996	312	65746	0.73	(0.64-0.83)	0.66	(0.58 - 0.75)	0.45	(0.40-0.50)	0.45	(0.41-0.51)	0.82	(0.73-0.92)	0.46	(0.41-0.52)	-1.30	0
- topotecan	1997	87	7885	-	-	1.19	(0.91 - 1.56)	1.06	(0.86-1.31)	1.07	(0.87-1.32)	1.92	(1.55-2.37)	1.12	(0.89-1.42)	-0.24	1
Type 2 topoisomerase inhibitors																	
Epipodophyllotoxines																	
- etoposide	1980	805	74977	-	-	0.97	(0.88 - 1.07)	1.03	(0.96-1.11)	1.04	(0.97-1.12)	1.87	(1.75-2.01)	1.13	(1.05-1.22)	-0.06	2
- teniposide	1976	10	435	-	-	2.88	(1.45 - 5.72)	2.24	(1.19-4.18)	2.26	(1.21-4.23)	4.05	(2.16-7.58)	2.72	(1.45-5.10)	0.03	6
Other type 2 topoisomerase inhibitors																	
- amsacrine	1981	15	457	-	-	0.81	(0.40 - 1.64)	3.22	(1.93-5.39)	3.26	(1.95-5.45)	5.84	(3.49-9.77)	4.08	(2.44-6.84)	0.74	5
- dactinomycin	1969	42	3680	-	-	1.75	(1.22 - 2.51)	1.10	(0.81-1.49)	1.11	(0.82-1.50)	1.99	(1.47-2.69)	1.32	(0.97-1.79)	-0.34	2
- pixantrone	2006	11	228	-	-	5.19	(2.58 - 10.44)	4.82	(2.63-8.83)	4.87	(2.66-8.92)	8.72	(4.76-15.99)	5.54	(2.94-10.45)	1.02	6
Others																	
KRAS inhibitor																	
- sotorasib	2019	17	2107	-	-	1.02	(0.53 - 1.98)	0.77	(0.48-1.25)	0.78	(0.48-1.26)	1.40	(0.87-2.26)	0.75	(0.44-1.30)	-1.13	0
Photosensitiser for photodynamic therapy																	
- padeliporfin	2013	0	8	-		-		-		-		-		-		-	-
Selective inhibitor of nuclear transport (SINE) compound																	
- selinexor	2015	40	4167	-	-	0.68	(0.37 - 1.25)	0.92	(0.67-1.26)	0.93	(0.68-1.27)	1.67	(1.22-2.28)	0.92	(0.62-1.36)	-0.60	1
EZH2 inhibitor																	
- tazemetostat	2017	2	899	-		-		-		-		-		-		-	-
PDE3 inhibitor																	
- anagrelide	1995	318	4003	6.66	(5.81-7.63)	7.52	(6.54 - 8.66)	8.25	(7.35-9.26)	8.34	(7.43-9.35)	14.87	(13.26-16.68)	9.60	(8.34-11.05)	2.75	7
Somatostatin analogue																	
- lanreotide	1997	56	13784	-	-	0.50	(0.37 - 0.67)	0.39	(0.30-0.50)	1.80	(1.08-3.00)	0.70	(0.54-0.91)	0.31	(0.22-0.43)	-1.75	1
- octreotide	1987	360	32327	-	-	1.18	(1.03 - 1.34)	1.07	(0.96-1.19)	1.92	(1.53-2.40)	1.94	(1.75-2.15)	0.90	(0.79-1.03)	-0.06	3
Inhibitor of adrenal cortex																	
- mitotane	1973	6	1127	-	-	0.37	(0.12 - 1.18)	0.51	(0.23-1.13)	0.51	(0.23-1.15)	0.92	(0.41-2.05)	0.24	(0.06-0.97)	-2.29	0
RAS GTPase inhibitor																	
- adagrasib	2021	2	282	-		-		-		-		-		-		-	-
Hypoxia-inducible factor-2 alpha (HIF-2) inhibitor																	
- belzutifan	2021	3	310	-		-		-		-		-		-		-	-
CXCR4 antagonist																	
- motixafortide	2016	0	6	-		-		-		-		-		-		-	-
Gamma secretase inhibitor																	
- nirogacestat	2019	0	37	-		-		-		-		-		-		-	-
Isocitrate Dehydrogenase 1 Inhibitors																	
- ivosidenib	2017	9	1263	-	-	0.73	(0.17 - 3.03)	0.68	(0.35-1.31)	0.69	(0.36-1.33)	1.24	(0.64-2.38)	0.90	(0.47-1.74)	-1.62	0
- olutasidenib	2017	0	71	-		-		-		-		-		-		-	-
Isocitrate Dehydrogenase 2 Inhibitors																	
- enasidenib	2018	12	2565	-	-	0.48	(0.24 - 0.96)	0.45	(0.25-0.79)	0.45	(0.26-0.80)	0.81	(0.46-1.43)	0.52	(0.28-0.96)	-2.05	0

Supplemental Table 3

Reporting Odds Ratio and their 95% confidence interval of serious events, cardiogenic shock and death of the anticancer drugs associated with cancer therapy-related cardiac dysfunction (CTRCD) in the primary population in VigiBase®. Seriousness was defined as the WHO definition (see manuscript). Multivariate analysis where cofactors were age, sex, concomitant medications of interest, concomitant cardiovascular conditions and coprescribed anticancer drugs (see Supplementary Data 1). Date of extraction was March, 1st, 2024. ROR: reporting odd-ratio. Yellow : analysis where the lower boundary of the 95%CI is ≥1 ; orange, ≥2 and dark orange ≥5.

		HF cases n=42828		Serious events n=37641 (all cancer drugs)				Cardiogenic shock n=2564 (all cancer drugs)				Death n=8833 (all cancer drugs)			
Drug	Year 1st report	(all cancer drugs)	Total reports	n, %		ROR	95%CI	n, %		ROR	95%CI	n, %		ROR	95%CI
Alkylating agents															
- mitomycin	1974	113	5438	40	35.4%	-	-	12	10.6%	3.15	(1.26-7.90)	9	8%	0.80	(0.32-1.99)
- carmustine	1971	78	4080	39	50%	-	-	10	12.8%	0.89	(0.24-3.34)	21	26.9%	2.72	(1.15-6.40)
- estramustine	1975	74	1811	50	67.6%	-	-	2	2.7%	-	-	12	16.2%	0.67	(0.27-1.64)
- busulfan	1968	160	9506	141	88.1%	0.82	(0.18-3.75)	23	14.4%	1.07	(0.54-2.15)	57	35.6%	1.60	(0.99-2.58)
- trabectedin	2004	99	2559	91	91.9%	0.93	(0.36-2.42)	5	5.1%	0.49	(0.17-1.38)	20	20.2%	1.80	(1.04-3.10)
ADT															
- bicalutamide	1995	364	15464	308	84.6%	1.17	(0.49-2.79)	23	6.3%	0.84	(0.44-1.61)	85	23.4%	1.01	(0.72-1.42)
- abiraterone	2008	578	39251	529	91.5%	0.45	(0.24-0.85)	23	4%	0.50	(0.25-1.00)	103	17.8%	0.76	(0.55-1.05)
- goserelin	1986	218	15662	159	72.9%	0.67	(0.33-1.37)	9	4.1%	1.06	(0.43-2.64)	43	19.7%	1.31	(0.84-2.04)
Anthracyclines															
- daunorubicin	1970	500	14663	399	79.8%	0.98	(0.45-2.13)	77	15.4%	1.13	(0.70-1.82)	125	25%	0.90	(0.63-1.28)
- doxorubicin	1974	2916	133357	2332	80%	1.30	(0.98-1.73)	245	8.4%	1.14	(0.89-1.47)	525	18%	0.73	(0.61-0.88)
- epirubicin	1985	749	35205	590	78.8%	1.18	(0.82-1.72)	33	4.4%	0.63	(0.36-1.11)	60	8%	0.66	(0.46-0.96)
- idarubicin	1987	175	8630	149	85.1%	0.95	(0.33-2.75)	18	10.3%	0.46	(0.21-0.99)	46	26.3%	1.20	(0.74-1.92)
- mitoxantrone	1984	529	7867	419	79.2%	1.09	(0.57-2.09)	40	7.6%	0.74	(0.46-1.19)	84	15.9%	0.50	(0.36-0.69)
Anti-aromatase															
- exemestane	2000	356	21889	335	94.1%	1.76	(0.86-3.59)	13	3.7%	0.67	(0.28-1.61)	71	19.9%	0.75	(0.50-1.10)
Antimetabolites															
- azacitidine	1978	352	19840	344	97.7%	1.76	(0.70-4.40)	24	6.8%	0.91	(0.53-1.56)	99	28.1%	1.25	(0.93-1.68)
- cytarabine	1970	1009	60400	829	82.2%	1.09	(0.56-2.13)	154	15.3%	1.39	(0.96-2.00)	283	28%	1.16	(0.89-1.52)
- fluorouracil	1969	1157	141176	940	81.2%	1.62	(1.01-2.59)	139	12%	1.95	(1.39-2.73)	164	14.2%	0.70	(0.53-0.92)
- gemcitabine	1994	1032	94322	774	75%	2.54	(1.41-4.58)	58	5.6%	0.46	(0.30-0.69)	212	20.5%	1.12	(0.91-1.37)
- clofarabine	2005	121	3405	116	95.9%	0.33	(0.10-1.08)	17	14%	0.73	(0.36-1.50)	73	60.3%	2.87	(1.75-4.72)
- fludarabine	1991	406	22897	359	88.4%	2.03	(0.66-6.20)	50	12.3%	1.15	(0.74-1.79)	162	39.9%	1.51	(1.11-2.06)
- pentostatin	1983	43	951	40	93%	-	-	7	16.3%	2.71	(0.92-8.01)	17	39.5%	1.55	(0.62-3.93)
CAR-T cells															
- axicabtagene ciloleucel	2018	63	5385	58	92.1%	0.83	(0.28-2.50)	9	14.3%	0.84	(0.34-2.07)	25	39.7%	2.26	(1.23-4.18)
- tisagenlecleucel	2015	58	3339	58	100%	-	-	10	17.2%	0.80	(0.33-1.91)	30	51.7%	2.86	(1.56-5.24)
Cytotoxic agents (not otherwise classified)															
- arsenic trioxide	1972	61	3083	56	91.8%	-	-	6	9.8%	0.98	(0.30-3.14)	14	23%	0.43	(0.19-0.99)
FLT3 inhibitor															
- midostaurin	2004	60	2802	58	96.7%	1.51	(0.34-6.71)	5	8.3%	0.37	(0.09-1.55)	15	25%	1.46	(0.74-2.90)
Immunomodulating agents															
- aldesleukin	1991	64	1856	37	57.8%	0.59	(0.07-4.93)	13	20.3%	2.96	(1.14-7.65)	12	18.8%	0.74	(0.33-1.70)
- thalidomide	1969	918	42908	811	88.3%	6.91	(1.68-28.40)	68	7.4%	0.52	(0.37-0.73)	415	45.2%	2.90	(2.41-3.49)

Drug	Year 1st report	HF cases n=42828 (all cancer drugs)	Total reports	Serious events n=37641 (all cancer drugs) n	%	ROR	95%CI	Cardiogenic shock n=2564 (all cancer drugs) n	%	ROR	95%CI	Death n=8833 (all cancer drugs) n	%	ROR	95%CI
Kinase inhibitors															
- alectinib	2014	79	6437	78	98.7%	4.07	(0.55-30.18)	2	2.5%	-	-	6	7.6%	0.41	(0.14-1.16)
- crizotinib	2010	181	13375	175	96.7%	1.82	(0.65-5.09)	5	2.8%	0.27	(0.10-0.74)	46	25.4%	1.66	(1.13-2.45)
- lorlatinib	2016	47	3817	46	97.9%	-	-	4	8.5%	-	-	9	19.1%	1.39	(0.63-3.08)
- bosutinib	2009	233	7525	223	95.7%	2.95	(1.18-7.35)	2	0.9%	-	-	22	9.4%	0.52	(0.32-0.87)
- dasatinib	2003	1415	35065	1332	94.1%	1.37	(0.99-1.91)	29	2%	0.42	(0.27-0.65)	83	5.9%	0.28	(0.21-0.37)
- imatinib	2002	1116	69004	1015	90.9%	0.99	(0.68-1.45)	36	3.2%	0.37	(0.23-0.60)	211	18.9%	0.92	(0.74-1.15)
- nilotinib	2006	510	32097	468	91.8%	0.91	(0.53-1.55)	28	5.5%	0.79	(0.45-1.40)	78	15.3%	0.72	(0.51-1.03)
- ponatinib	2011	214	8780	210	98.1%	1.72	(0.53-5.56)	14	6.5%	0.42	(0.19-0.93)	67	31.3%	1.79	(1.21-2.64)
- ibrutinib	2013	1312	68915	1285	97.9%	1.84	(1.05-3.23)	49	3.7%	0.55	(0.37-0.83)	234	17.8%	0.76	(0.62-0.93)
- osimertinib	2014	436	19739	407	93.3%	2.38	(1.25-4.54)	13	3%	0.19	(0.09-0.42)	84	19.3%	1.51	(1.10-2.07)
- gilteritinib	2017	24	1160	24	100%	-	-	2	8.3%	-	-	10	41.7%	4.76	(1.84-12.31)
- ruxolitinib	2012	626	58126	610	97.4%	1.71	(0.74-3.98)	34	5.4%	0.55	(0.32-0.93)	196	31.3%	1.93	(1.48-2.51)
- cobimetinib	2012	78	3075	69	88.5%	0.65	(0.18-2.39)	3	3.8%	-	-	11	14.1%	0.48	(0.19-1.22)
- selumetinib	2010	19	986	17	89.5%	0.68	(0.14-3.38)	0	0%	-	-	0	0%	-	-
- trametinib	2011	383	19923	316	82.5%	0.44	(0.19-1.05)	18	4.7%	1.42	(0.55-3.65)	58	15.1%	1.00	(0.54-1.87)
- dabrafenib	2011	347	18325	289	83.3%	0.91	(0.37-2.25)	11	3.2%	0.39	(0.13-1.18)	49	14.1%	0.75	(0.38-1.46)
- pralsetinib	2020	16	708	16	100%	-	-	1	6.2%	-	-	2	12.5%	-	-
- entrectinib	2017	35	758	34	97.1%	-	-	1	2.9%	-	-	4	11.4%	-	-
- lenvatinib	2013	285	19204	277	97.2%	2.39	(1.03-5.56)	7	2.5%	0.34	(0.13-0.86)	27	9.5%	0.44	(0.28-0.68)
- nintedanib	2008	371	27369	367	98.9%	6.70	(1.63-27.47)	24	6.5%	0.63	(0.38-1.06)	127	34.2%	1.91	(1.46-2.50)
- pazopanib	2005	382	28536	350	91.6%	0.94	(0.54-1.64)	26	6.8%	0.85	(0.50-1.46)	69	18.1%	1.05	(0.75-1.46)
- sunitinib	2003	909	42181	847	93.2%	1.06	(0.71-1.59)	46	5.1%	0.66	(0.44-1.01)	167	18.4%	0.82	(0.64-1.03)
Mitotic spindle poisons															
- vinblastine	1969	145	8753	104	71.7%	0.76	(0.14-4.03)	18	12.4%	1.15	(0.36-3.61)	37	25.5%	1.85	(0.78-4.37)
- vinorelbine	1990	348	18392	222	63.8%	0.90	(0.48-1.70)	19	5.5%	1.03	(0.51-2.06)	70	20.1%	1.94	(1.30-2.92)
Monoclonal antibodies															
- ibritumomab tiuxetan	2000	20	899	19	95%	-	-	2	10%	-	-	7	35%	0.57	(0.17-1.92)
- basiliximab	1999	107	6453	88	82.2%	2.36	(0.31-18.17)	17	15.9%	1.92	(0.93-3.95)	19	17.8%	0.53	(0.29-0.97)
- brentuximab vedotin	2011	90	7933	86	95.6%	1.06	(0.31-3.58)	10	11.1%	1.09	(0.50-2.35)	30	33.3%	2.16	(1.23-3.78)
- gemtuzumab ozogamicin	2001	103	2712	91	88.3%	1.79	(0.23-14.01)	10	9.7%	0.88	(0.39-1.96)	39	37.9%	1.47	(0.88-2.45)
- alemtuzumab	1995	195	24149	181	92.8%	-	-	26	13.3%	1.07	(0.61-1.88)	68	34.9%	1.14	(0.77-1.70)
- dinutuximab	2015	10	318	9	90%	0.18	(0.01-2.42)	2	20%	-	-	2	20%	-	-
- trastuzumab	1999	4229	60951	3234	76.5%	0.43	(0.35-0.53)	62	1.5%	0.31	(0.21-0.46)	177	4.2%	0.23	(0.19-0.29)
- trastuzumab emtansine	2010	217	8022	162	74.7%	0.54	(0.35-0.82)	0	0%	-	-	18	8.3%	1.19	(0.65-2.20)
- trastuzumab deruxtecan	2019	89	7006	55	61.8%	0.29	(0.14-0.58)	0	0%	-	-	12	13.5%	2.32	(1.04-5.19)
- bevacizumab	2000	1212	96520	1146	94.6%	1.18	(0.80-1.75)	60	5%	0.42	(0.28-0.62)	229	18.9%	0.64	(0.52-0.79)
- ramucirumab	2010	78	8917	73	93.6%	0.62	(0.21-1.83)	4	5.1%	-	-	6	7.7%	0.19	(0.04-0.83)
mTOR inhibitors															
- everolimus	2002	684	53092	642	93.9%	0.84	(0.51-1.40)	33	4.8%	0.83	(0.52-1.31)	158	23.1%	1.49	(1.17-1.91)
- temsirolimus	2003	84	4053	79	94%	1.76	(0.24-13.05)	4	4.8%	-	-	22	26.2%	1.07	(0.56-2.06)
Proteasom inhibitors															
- bortezomib	2000	1396	65793	1343	96.2%	1.44	(0.89-2.32)	102	7.3%	1.15	(0.87-1.51)	347	24.9%	0.79	(0.67-0.94)
- carfilzomib	2009	1046	20700	981	93.8%	1.09	(0.71-1.68)	42	4%	0.74	(0.49-1.11)	162	15.5%	0.62	(0.49-0.79)
Topoisomerase inhibitors															
- teniposide	1976	10	435	8	80%	-	-	1	10%	-	-	2	20%	-	-
- dactinomycin	1969	42	3680	35	83.3%	1.25	(0.25-6.26)	5	11.9%	0.99	(0.30-3.33)	12	28.6%	1.51	(0.58-3.93)
- pixantrone	2006	11	228	10	90.9%	-	-	1	9.1%	-	-	0	0%	-	-
Others															
- anagrelide	1995	318	4003	265	83.3%	2.53	(0.91-7.01)	12	3.8%	0.58	(0.27-1.24)	28	8.8%	0.30	(0.18-0.50)
- octreotide	1987	360	32327	333	92.5%	-	-	34	9.4%	1.19	(0.68-2.10)	124	34.4%	1.44	(1.04-1.99)

Supplemental Table 4

Characteristics of cancer therapy-related cardiac dysfunction (CTRCD) cases of the anticancer drugs associated with CTRCD in either primary analysis or multivariate sensitivity analysis in Vigibase®. Date of extraction was March, 1st, 2024. DVT: deep venous thrombosis, PE: pulmonary embolism, TdP: torsade de pointes.

						Concurrent adverse events																			
	No	Male		Age ≥65 ans		Acute coronary syndrome		Hypertension		DVT and/or PE		Supraventricular tachyarrhythmias		Ventricular tachyarrhythmias		TdP/QT prolongation		Conduction disorders		Valve disorders		Noninfectious myocarditis		Hyperthyroidism	
Alkylating agents																									
mitomycin	113	33/100	33.0%	31/90	34.4%	5/113	4.4%	5/113	4.4%	4/113	3.5%	3/113	2.7%	-	-	-	-	1/113	0.9%	1/113	0.9%	3/113	2.7%	-	-
carmustine	78	14/69	20.3%	10/59	16.9%	1/78	1.3%	1/78	1.3%	3/78	3.8%	6/78	7.7%	3/78	3.8%	3/78	3.8%	1/78	1.3%	1/78	1.3%	-	-	-	-
estramustine	74	72/73	98.6%	50/58	86.2%	7/74	9.5%	9/74	12.2%	3/74	4.1%	3/74	4.1%	-	-	-	-	1/74	1.4%	-	-	-	-	-	-
busulfan	160	78/141	55.3%	7/117	6.0%	8/160	5.0%	8/160	5.0%	21/160	13.1%	9/160	5.6%	2/160	1.2%	2/160	1.2%	2/160	1.2%	4/160	2.5%	5/160	3.1%	-	-
trabectedin	99	37/95	38.9%	44/81	54.3%	4/99	4.0%	1/99	1.0%	2/99	2.0%	7/99	7.1%	1/99	1.0%	2/99	2.0%	-	-	1/99	1.0%	-	-	-	-
ADT																									
bicalutamide	364	358/359	99.7%	269/298	90.3%	34/364	9.3%	29/364	8.0%	12/364	3.3%	23/364	6.3%	11/364	3.0%	8/364	2.2%	15/364	4.1%	19/364	5.2%	-	-	1/364	0.3%
abiraterone	578	564/566	99.6%	396/423	93.6%	25/578	4.3%	30/578	5.2%	7/578	1.2%	54/578	9.3%	14/578	2.4%	11/578	1.9%	2/578	0.3%	9/578	1.6%	-	-	-	-
goserelin	218	168/214	78.5%	130/183	71.0%	16/218	7.3%	23/218	10.6%	5/218	2.3%	12/218	5.5%	1/218	0.5%	2/218	0.9%	4/218	1.8%	9/218	4.1%	-	-	-	-
Anthracyclines																									
daunorubicin	500	202/468	43.2%	92/359	25.6%	24/500	4.8%	29/500	5.8%	9/500	1.8%	50/500	10.0%	8/500	1.6%	12/500	2.4%	3/500	0.6%	7/500	1.4%	1/500	0.2%	4/500	0.8%
doxorubicin	2916	769/2,584	29.8%	704/2,145	32.8%	119/2,916	4.1%	97/2,916	3.3%	110/2,916	3.8%	190/2,916	6.5%	49/2,916	1.7%	22/2,916	0.8%	43/2,916	1.5%	100/2,916	3.4%	19/2,916	0.7%	5/2,916	0.2%
epirubicin	749	44/712	6.2%	160/610	26.2%	14/749	1.9%	8/749	1.1%	13/749	1.7%	23/749	3.1%	5/749	0.7%	2/749	0.3%	7/749	0.9%	19/749	2.5%	9/749	1.2%	1/749	0.1%
idarubicin	175	84/162	51.9%	34/154	22.1%	11/175	6.3%	2/175	1.1%	2/175	1.1%	20/175	11.4%	5/175	2.9%	3/175	1.7%	1/175	0.6%	9/175	5.1%	9/175	5.1%	-	-
mitoxantrone	529	175/494	35.4%	71/378	18.8%	13/529	2.5%	11/529	2.1%	20/529	3.8%	18/529	3.4%	7/529	1.3%	7/529	1.3%	9/529	1.7%	36/529	6.8%	4/529	0.8%	1/529	0.2%
Anti-aromatase																									
exemestane	356	3/350	0.9%	149/259	57.5%	21/356	5.9%	44/356	12.4%	21/356	5.9%	41/356	11.5%	5/356	1.4%	3/356	0.8%	10/356	2.8%	31/356	8.7%	1/356	0.3%	2/356	0.6%
Antimetabolites																									
azacitidine	352	203/318	63.8%	213/270	78.9%	13/352	3.7%	12/352	3.4%	9/352	2.6%	27/352	7.7%	5/352	1.4%	6/352	1.7%	1/352	0.3%	6/352	1.7%	1/352	0.3%	-	-
cytarabine	1009	450/938	48.0%	229/777	29.5%	45/1,009	4.5%	52/1,009	5.2%	29/1,009	2.9%	107/1,009	10.6%	24/1,009	2.4%	30/1,009	3.0%	9/1,009	0.9%	22/1,009	2.2%	11/1,009	1.1%	5/1,009	0.5%
fluorouracil	1157	451/1,104	40.9%	418/965	43.3%	67/1,157	5.8%	37/1,157	3.2%	46/1,157	4.0%	69/1,157	6.0%	25/1,157	2.2%	16/1,157	1.4%	17/1,157	1.5%	40/1,157	3.5%	30/1,157	2.6%	2/1,157	0.2%
gemcitabine	1032	459/969	47.4%	450/851	52.9%	70/1,032	6.8%	72/1,032	7.0%	51/1,032	4.9%	64/1,032	6.2%	8/1,032	0.8%	3/1,032	0.3%	11/1,032	1.1%	28/1,032	2.7%	2/1,032	0.2%	1/1,032	0.1%
clofarabine	121	51/110	46.4%	37/93	39.8%	10/121	8.3%	8/121	6.6%	7/121	5.8%	21/121	17.4%	3/121	2.5%	5/121	4.1%	2/121	1.7%	7/121	5.8%	1/121	0.8%	2/121	1.7%
fludarabine	406	194/359	54.0%	96/315	30.5%	21/406	5.2%	25/406	6.2%	31/406	7.6%	36/406	8.9%	2/406	0.5%	3/406	0.7%	2/406	0.5%	12/406	3.0%	11/406	2.7%	-	-
pentostatin	43	19/35	54.3%	13/29	44.8%	4/43	9.3%	3/43	7.0%	1/43	2.3%	7/43	16.3%	-	-	-	-	1/43	2.3%	1/43	2.3%	1/43	2.3%	-	-
CAR-T cells																									
axicabtagene ciloleucel	63	36/60	60.0%	29/57	50.9%	1/63	1.6%	2/63	3.2%	4/63	6.3%	11/63	17.5%	2/63	3.2%	2/63	3.2%	-	-	-	-	-	-	-	-
tisagenlecleucel	58	31/56	55.4%	20/56	35.7%	2/58	3.4%	5/58	8.6%	1/58	1.7%	2/58	3.4%	2/58	3.4%	2/58	3.4%	-	-	4/58	6.9%	1/58	1.7%	-	-
Cytotoxic agents (not otherwise classified)																									
arsenic trioxide	61	29/53	54.7%	30/50	60.0%	2/61	3.3%	3/61	4.9%	1/61	1.6%	8/61	13.1%	4/61	6.6%	11/61	18.0%	1/61	1.6%	10/61	16.4%	1/61	1.6%	-	-
FLT3 inhibitor																									
midostaurin	60	24/55	43.6%	21/51	41.2%	1/60	1.7%	4/60	6.7%	1/60	1.7%	3/60	5.0%	2/60	3.3%	5/60	8.3%	1/60	1.7%	3/60	5.0%	2/60	3.3%	-	-
Immunomodulating agents																									
aldesleukin	64	38/60	63.3%	14/54	25.9%	9/64	14.1%	2/64	3.1%	5/64	7.8%	10/64	15.6%	2/64	3.1%	1/64	1.6%	1/64	1.6%	2/64	3.1%	3/64	4.7%	-	-
thalidomide	918	532/885	60.1%	541/748	72.3%	79/918	8.6%	58/918	6.3%	70/918	7.6%	88/918	9.6%	21/918	2.3%	14/918	1.5%	36/918	3.9%	52/918	5.7%	2/918	0.2%	5/918	0.5%
Kinase inhibitors																									
alectinib	79	28/76	36.8%	25/49	51.0%	2/79	2.5%	-	-	1/79	1.3%	3/79	3.8%	1/79	1.3%	2/79	2.5%	-	-	-	-	-	-	-	-
crizotinib	181	69/163	42.3%	101/148	68.2%	3/181	1.7%	3/181	1.7%	9/181	5.0%	4/181	2.2%	-	-	7/181	3.9%	1/181	0.6%	1/181	0.6%	-	-	-	-
lorlatinib	47	23/44	52.3%	16/40	40.0%	-	-	1/47	2.1%	2/47	4.3%	3/47	6.4%	1/47	2.1%	1/47	2.1%	-	-	-	-	-	-	-	-
bosutinib	233	108/211	51.2%	118/182	64.8%	8/233	3.4%	4/233	1.7%	3/233	1.3%	9/233	3.9%	-	-	-	-	-	-	2/233	0.9%	1/233	0.4%	-	-
dasatinib	1415	670/1,362	49.2%	592/988	59.9%	33/1,415	2.3%	30/1,415	2.1%	7/1,415	0.5%	42/1,415	3.0%	4/1,415	0.3%	8/1,415	0.6%	6/1,415	0.4%	14/1,415	1.0%	2/1,415	0.1%	-	-
imatinib	1116	558/1,046	53.3%	458/737	62.1%	48/1,116	4.3%	40/1,116	3.6%	15/1,116	1.3%	80/1,116	7.2%	15/1,116	1.3%	8/1,116	0.7%	10/1,116	0.9%	44/1,116	3.9%	5/1,116	0.4%	1/1,116	0.1%
nilotinib	510	269/466	57.7%	193/300	64.3%	51/510	10.0%	44/510	8.6%	9/510	1.8%	44/510	8.6%	10/510	2.0%	17/510	3.3%	9/510	1.8%	34/510	6.7%	-	-	1/510	0.2%
ponatinib	214	105/187	56.1%	83/140	59.3%	19/214	8.9%	19/214	8.9%	16/214	7.5%	29/214	13.6%	1/214	0.5%	1/214	0.5%	2/214	0.9%	10/214	4.7%	1/214	0.5%	-	-
ibrutinib	1312	830/1,252	66.3%	692/801	86.4%	47/1,312	3.6%	60/1,312	4.6%	14/1,312	1.1%	318/1,312	24.2%	19/1,312	1.4%	12/1,312	0.9%	5/1,312	0.4%	25/1,312	1.9%	2/1,312	0.2%	-	-
osimertinib	436	146/417	35.0%	227/305	74.4%	9/436	2.1%	4/436	0.9%	7/436	1.6%	22/436	5.0%	8/436	1.8%	18/436	4.1%	2/436	0.5%	8/436	1.8%	2/436	0.5%	-	-
gilteritinib	24	10/22	45.5%	9/21	42.9%	2/24	8.3%	1/24	4.2%	-	-	1/24	4.2%	-	-	2/24	8.3%	-	-	-	-	1/24	4.2%	-	-
ruxolitinib	626	232/393	59.0%	262/317	82.6%	33/626	5.3%	40/626	6.4%	21/626	3.4%	48/626	7.7%	4/626	0.6%	3/626	0.5%	7/626	1.1%	27/626	4.3%	1/626	0.2%	2/626	0.3%
cobimetinib	78	35/73	47.9%	31/60	51.7%	5/78	6.4%	4/78	5.1%	2/78	2.6%	7/78	9.0%	-	-	3/78	3.8%	1/78	1.3%	3/78	3.8%	1/78	1.3%	-	-
selumetinib	19	14/17	82.4%	1/15	6.7%	-	-	1/19	5.3%	1/19	5.3%	2/19	10.5%	-	-	-	-	1/19	5.3%	-	-	-	-	-	-

												Concurrent adverse events													
	No	Male		Age ≥65 ans		Acute coronary syndrome		Hypertension		DVT and/or PE		Supraventricular tachyarrhythmias		Ventricular tachyarrhythmias		TdP/QT prolongation		Conduction disorders		Valve disorders		Noninfectious myocarditis		Hyperthyroidism	
trametinib	383	220/361	60.9%	139/285	48.8%	14/383	3.7%	9/383	2.3%	12/383	3.1%	30/383	7.8%	3/383	0.8%	7/383	1.8%	4/383	1.0%	11/383	2.9%	3/383	0.8%	-	-
dabrafenib	347	214/328	65.2%	135/256	52.7%	10/347	2.9%	8/347	2.3%	13/347	3.7%	26/347	7.5%	2/347	0.6%	5/347	1.4%	4/347	1.2%	10/347	2.9%	3/347	0.9%	-	-
pralsetinib	16	6/16	37.5%	6/12	50.0%	-	-	2/16	12.5%	-	-	1/16	6.2%	-	-	-	-	-	-	-	-	-	-	-	-
entrectinib	35	8/34	23.5%	14/27	51.9%	1/35	2.9%	-	-	-	-	-	-	-	-	-	-	-	-	2/35	5.7%	1/35	2.9%	-	-
lenvatinib	285	127/282	45.0%	150/242	62.0%	6/285	2.1%	72/285	25.3%	4/285	1.4%	7/285	2.5%	1/285	0.4%	5/285	1.8%	2/285	0.7%	2/285	0.7%	-	-	-	-
nintedanib	371	240/361	66.5%	244/299	81.6%	20/371	5.4%	12/371	3.2%	11/371	3.0%	19/371	5.1%	2/371	0.5%	1/371	0.3%	5/371	1.3%	8/371	2.2%	-	-	-	-
pazopanib	382	194/352	55.1%	152/271	56.1%	12/382	3.1%	51/382	13.4%	6/382	1.6%	16/382	4.2%	4/382	1.0%	6/382	1.6%	6/382	1.6%	9/382	2.4%	1/382	0.3%	1/382	0.3%
sunitinib	909	517/834	62.0%	377/627	60.1%	35/909	3.9%	68/909	7.5%	12/909	1.3%	29/909	3.2%	6/909	0.7%	5/909	0.6%	4/909	0.4%	14/909	1.5%	2/909	0.2%	4/909	0.4%
Mitotic spindle poisons																									
vinblastine	145	78/132	59.1%	36/120	30.0%	8/145	5.5%	8/145	5.5%	5/145	3.4%	9/145	6.2%	1/145	0.7%	1/145	0.7%	2/145	1.4%	5/145	3.4%	2/145	1.4%	-	-
vinorelbine	348	97/323	30.0%	113/272	41.5%	18/348	5.2%	20/348	5.7%	20/348	5.7%	22/348	6.3%	7/348	2.0%	3/348	0.9%	-	-	13/348	3.7%	1/348	0.3%	2/348	0.6%
Monoclonal antibodies																									
ibritumomab tiuxetan	20	15/17	88.2%	7/18	38.9%	-	-	-	-	2/20	10.0%	5/20	25.0%	-	-	-	-	1/20	5.0%	4/20	20.0%	-	-	-	-
basiliximab	107	71/101	70.3%	16/96	16.7%	11/107	10.3%	10/107	9.3%	8/107	7.5%	7/107	6.5%	2/107	1.9%	-	-	1/107	0.9%	1/107	0.9%	1/107	0.9%	-	-
brentuximab vedotin	90	49/84	58.3%	25/69	36.2%	3/90	3.3%	-	-	4/90	4.4%	10/90	11.1%	-	-	-	-	-	-	-	-	-	-	-	-
gemtuzumab ozogamicin	103	39/100	39.0%	30/81	37.0%	8/103	7.8%	8/103	7.8%	14/103	13.6%	9/103	8.7%	1/103	1.0%	2/103	1.9%	2/103	1.9%	4/103	3.9%	1/103	1.0%	1/103	1.0%
alemtuzumab	195	67/164	40.9%	35/150	23.3%	16/195	8.2%	20/195	10.3%	10/195	5.1%	14/195	7.2%	4/195	2.1%	4/195	2.1%	4/195	2.1%	1/195	0.5%	4/195	2.1%	5/195	2.6%
dinutuximab	10	5/8	62.5%	0/7	0.0%	-	-	2/10	20.0%	1/10	10.0%	-	-	-	-	-	-	-	-	-	-	-	-	-	-
trastuzumab	4229	106/3,785	2.8%	794/2,667	29.8%	48/4,229	1.1%	68/4,229	1.6%	53/4,229	1.3%	65/4,229	1.5%	22/4,229	0.5%	11/4,229	0.3%	22/4,229	0.5%	55/4,229	1.3%	17/4,229	0.4%	5/4,229	0.1%
trastuzumab emtansine	217	6/199	3.0%	39/160	24.4%	4/217	1.8%	5/217	2.3%	3/217	1.4%	2/217	0.9%	-	-	-	-	1/217	0.5%	1/217	0.5%	-	-	-	-
trastuzumab deruxtecan	89	6/77	7.8%	19/50	38.0%	2/89	2.2%	1/89	1.1%	1/89	1.1%	-	-	-	-	-	-	-	-	-	-	-	-	-	-
bevacizumab	1212	369/1,108	33.3%	424/865	49.0%	73/1,212	6.0%	141/1,212	11.6%	72/1,212	5.9%	76/1,212	6.3%	20/1,212	1.7%	12/1,212	1.0%	11/1,212	0.9%	33/1,212	2.7%	3/1,212	0.2%	2/1,212	0.2%
ramucirumab	78	48/69	69.6%	42/57	73.7%	1/78	1.3%	8/78	10.3%	2/78	2.6%	2/78	2.6%	-	-	1/78	1.3%	3/78	3.8%	2/78	2.6%	-	-	-	-
mTOR inhibitors																									
everolimus	684	269/658	40.9%	247/449	55.0%	26/684	3.8%	61/684	8.9%	20/684	2.9%	45/684	6.6%	5/684	0.7%	5/684	0.7%	2/684	0.3%	41/684	6.0%	-	-	1/684	0.1%
temsirolimus	84	46/76	60.5%	28/52	53.8%	7/84	8.3%	7/84	8.3%	2/84	2.4%	3/84	3.6%	-	-	-	-	1/84	1.2%	2/84	2.4%	-	-	-	-
Proteasom inhibitors																									
bortezomib	1396	675/1,274	53.0%	708/1,093	64.8%	67/1,396	4.8%	65/1,396	4.7%	49/1,396	3.5%	157/1,396	11.2%	19/1,396	1.4%	17/1,396	1.2%	20/1,396	1.4%	57/1,396	4.1%	2/1,396	0.1%	5/1,396	0.4%
carfilzomib	1046	507/895	56.6%	499/729	68.4%	50/1,046	4.8%	86/1,046	8.2%	33/1,046	3.2%	63/1,046	6.0%	8/1,046	0.8%	12/1,046	1.1%	12/1,046	1.1%	10/1,046	1.0%	2/1,046	0.2%	-	-
Topoisomerase inhibitors																									
teniposide	10	5/10	50.0%	1/9	11.1%	-	-	1/10	10.0%	-	-	1/10	10.0%	1/10	10.0%	-	-	1/10	10.0%	1/10	10.0%	-	-	-	-
dactinomycin	42	14/35	40.0%	0/36	0.0%	-	-	-	-	6/42	14.3%	2/42	4.8%	-	-	-	-	-	-	4/42	9.5%	-	-	1/42	2.4%
pixantrone	11	6/9	66.7%	6/9	66.7%	1/11	9.1%	-	-	-	-	1/11	9.1%	1/11	9.1%	1/11	9.1%	-	-	-	-	-	-	-	-
Others																									
anagrelide	318	141/302	46.7%	196/266	73.7%	12/318	3.8%	9/318	2.8%	1/318	0.3%	27/318	8.5%	5/318	1.6%	2/318	0.6%	2/318	0.6%	9/318	2.8%	-	-	-	-
octreotide	360	150/346	43.4%	150/264	56.8%	21/360	5.8%	84/360	23.3%	10/360	2.8%	27/360	7.5%	9/360	2.5%	7/360	1.9%	6/360	1.7%	42/360	11.7%	-	-	-	-

Supplemental Table 5

Characteristics of concomitant medications in cases of cancer therapy-related cardiac dysfunction (CTRCD) associated with the anticancer drugs associated to CTRCD overreporting in Vigibase® in either primary analysis or sensitivity multivariate analysis. The extraction date was March 1st, 2024. RAAS, renin angiotensin aldosterone system.

		Concurrent medications					
	No	RAAS blockers		Beta-blockers		Diuretics	
Alkylating agents							
mitomycin	113	3/113	2.7%	6/113	5.3%	8/113	7.1%
carmustine	78	-	-	2/78	2.6%	5/78	6.4%
estramustine	74	12/74	16.2%	12/74	16.2%	12/74	16.2%
busulfan	160	6/160	3.8%	11/160	6.9%	14/160	8.8%
trabectedin	99	15/99	15.2%	11/99	11.1%	10/99	10.1%
ADT							
bicalutamide	364	112/364	30.8%	126/364	34.6%	133/364	36.5%
abiraterone	578	81/578	14.0%	87/578	15.1%	101/578	17.5%
goserelin	218	47/218	21.6%	34/218	15.6%	44/218	20.2%
Anthracyclines							
daunorubicin	500	19/500	3.8%	14/500	2.8%	35/500	7.0%
doxorubicin	2916	179/2,916	6.1%	184/2,916	6.3%	200/2,916	6.9%
epirubicin	749	45/749	6.0%	36/749	4.8%	28/749	3.7%
idarubicin	175	8/175	4.6%	10/175	5.7%	18/175	10.3%
mitoxantrone	529	31/529	5.9%	26/529	4.9%	36/529	6.8%
Anti-aromatase							
exemestane	356	93/356	26.1%	90/356	25.3%	81/356	22.8%
Antimetabolites							
azacitidine	352	52/352	14.8%	60/352	17.0%	88/352	25.0%
cytarabine	1009	54/1,009	5.4%	65/1,009	6.4%	92/1,009	9.1%
fluorouracil	1157	108/1,157	9.3%	104/1,157	9.0%	109/1,157	9.4%
gemcitabine	1032	97/1,032	9.4%	91/1,032	8.8%	134/1,032	13.0%
clofarabine	121	15/121	12.4%	20/121	16.5%	26/121	21.5%
fludarabine	406	23/406	5.7%	35/406	8.6%	45/406	11.1%
pentostatin	43	6/43	14.0%	9/43	20.9%	10/43	23.3%
CAR-T cells							
axicabtagene ciloleucel	63	6/63	9.5%	5/63	7.9%	5/63	7.9%
tisagenlecleucel	58	2/58	3.4%	5/58	8.6%	4/58	6.9%
Cytotoxic agents (not otherwise classified)							
arsenic trioxide	61	5/61	8.2%	8/61	13.1%	14/61	23.0%
FLT3 inhibitor							
midostaurin	60	7/60	11.7%	9/60	15.0%	9/60	15.0%
Immunomodulating agents							
aldesleukin	64	1/64	1.6%	4/64	6.2%	4/64	6.2%
thalidomide	918	107/918	11.7%	144/918	15.7%	169/918	18.4%
Kinase inhibitors							
alectinib	79	10/79	12.7%	8/79	10.1%	12/79	15.2%
crizotinib	181	18/181	9.9%	10/181	5.5%	18/181	9.9%
lorlatinib	47	4/47	8.5%	5/47	10.6%	6/47	12.8%
bosutinib	233	29/233	12.4%	46/233	19.7%	45/233	19.3%
dasatinib	1415	151/1,415	10.7%	174/1,415	12.3%	197/1,415	13.9%
imatinib	1116	119/1,116	10.7%	143/1,116	12.8%	179/1,116	16.0%
nilotinib	510	61/510	12.0%	62/510	12.2%	71/510	13.9%
ponatinib	214	38/214	17.8%	44/214	20.6%	43/214	20.1%
ibrutinib	1312	231/1,312	17.6%	305/1,312	23.2%	301/1,312	22.9%
osimertinib	436	30/436	6.9%	44/436	10.1%	34/436	7.8%

		Concurrent medications					
	No	RAAS blockers		Beta-blockers		Diuretics	
gilteritinib	24	2/24	8.3%	1/24	4.2%	-	-
ruxolitinib	626	138/626	22.0%	228/626	36.4%	213/626	34.0%
cobimetinib	78	11/78	14.1%	13/78	16.7%	10/78	12.8%
selumetinib	19	2/19	10.5%	1/19	5.3%	-	-
trametinib	383	44/383	11.5%	39/383	10.2%	28/383	7.3%
dabrafenib	347	45/347	13.0%	36/347	10.4%	26/347	7.5%
pralsetinib	16	1/16	6.2%	1/16	6.2%	1/16	6.2%
entrectinib	35	2/35	5.7%	4/35	11.4%	3/35	8.6%
lenvatinib	285	49/285	17.2%	44/285	15.4%	36/285	12.6%
nintedanib	371	55/371	14.8%	47/371	12.7%	64/371	17.3%
pazopanib	382	46/382	12.0%	49/382	12.8%	39/382	10.2%
sunitinib	909	140/909	15.4%	145/909	16.0%	126/909	13.9%
Mitotic spindle poisons							
vinblastine	145	10/145	6.9%	7/145	4.8%	10/145	6.9%
vinorelbine	348	35/348	10.1%	26/348	7.5%	30/348	8.6%
Monoclonal antibodies							
ibritumomab tiuxetan	20	2/20	10.0%	2/20	10.0%	3/20	15.0%
basiliximab	107	9/107	8.4%	14/107	13.1%	16/107	15.0%
brentuximab vedotin	90	7/90	7.8%	10/90	11.1%	9/90	10.0%
gemtuzumab ozogamicin	103	7/103	6.8%	6/103	5.8%	12/103	11.7%
alemtuzumab	195	5/195	2.6%	10/195	5.1%	13/195	6.7%
dinutuximab	10	-	-	-	-	1/10	10.0%
trastuzumab	4229	243/4,229	5.7%	196/4,229	4.6%	166/4,229	3.9%
trastuzumab emtansine	217	24/217	11.1%	24/217	11.1%	13/217	6.0%
trastuzumab deruxtecan	89	6/89	6.7%	9/89	10.1%	6/89	6.7%
bevacizumab	1212	133/1,212	11.0%	109/1,212	9.0%	126/1,212	10.4%
ramucirumab	78	12/78	15.4%	2/78	2.6%	11/78	14.1%
mTOR inhibitors							
everolimus	684	107/684	15.6%	137/684	20.0%	119/684	17.4%
temsirolimus	84	12/84	14.3%	15/84	17.9%	15/84	17.9%
Proteasom inhibitors							
bortezomib	1396	196/1,396	14.0%	239/1,396	17.1%	264/1,396	18.9%
carfilzomib	1046	85/1,046	8.1%	90/1,046	8.6%	83/1,046	7.9%
Topoisomerase inhibitors							
teniposide	10	-	-	-	-	-	-
dactinomycin	42	-	-	-	-	-	-
pixantrone	11	1/11	9.1%	1/11	9.1%	-	-
Others							
anagrelide	318	60/318	18.9%	54/318	17.0%	67/318	21.1%
octreotide	360	78/360	21.7%	64/360	17.8%	98/360	27.2%

Supplemental Table 6

Time to onset (TTO) from treatment initiation for the anticancer drugs associated with cancer therapy-related cardiac dysfunction (CTRCD) overreporting in Vigibase® in our multivariate sensibility analysis. Only anticancer drugs with >4 reports with TTO available are displayed. Date of extraction was March, 1st, 2024.

	cases, n	TTO, median (interquartile), in days
abiraterone	134	63.5 (25.2-165.5)
aldesleukin	24	10.0 (3.8-28.8)
alectinib	14	41.0 (21.8-78.2)
alemtuzumab	35	23.0 (3.0-136.5)
anagrelide	110	207.0 (55.5-489.2)
arsenic trioxide	11	29.0 (10.0-44.5)
axicabtagene ciloleucel	10	7.0 (2.0-15.0)
azacitidine	131	37.0 (13.0-129.5)
basiliximab	32	2.5 (0.0-27.2)
bevacizumab	367	98.0 (29.0-234.5)
bicalutamide	42	39.5 (8.5-145.5)
bortezomib	310	25.0 (7.2-71.5)
bosutinib	33	67.0 (31.0-421.0)
brentuximab vedotin	24	36.0 (8.8-130.8)
busulfan	26	23.0 (11.2-44.2)
carfilzomib	277	22.0 (9.0-77.0)
carmustine	22	8.0 (0.2-25.8)
clofarabine	23	15.0 (8.5-41.5)
cobimetinib	32	67.0 (19.0-176.5)
crizotinib	41	28.0 (14.0-76.0)
cytarabine	181	16.0 (6.0-33.0)
dabrafenib	110	94.5 (36.5-206.0)
dasatinib	224	70.0 (14.0-424.2)
daunorubicin	113	21.0 (10.0-55.0)
doxorubicin	637	125.0 (30.0-261.0)
entrectinib	9	99.0 (60.0-328.0)
epirubicin	250	177.0 (54.0-349.8)
estramustine	22	31.5 (17.2-54.2)
everolimus	182	94.0 (44.2-218.2)
exemestane	42	242.5 (47.0-671.5)
fludarabine	72	15.0 (7.0-44.2)
fluorouracil	300	19.5 (3.0-140.2)
gemcitabine	338	49.0 (8.0-122.8)
gemtuzumab ozogamicin	17	11.0 (1.0-19.0)
gilteritinib	7	27.0 (12.5-38.0)
goserelin	35	245.0 (73.5-660.5)
ibrutinib	120	172.0 (54.5-414.0)
idarubicin	51	17.0 (9.5-66.5)
imatinib	161	70.0 (30.0-376.0)
lenvatinib	51	46.0 (17.0-90.5)
lorlatinib	14	147.0 (90.8-201.2)
midostaurin	26	22.0 (10.0-62.8)
mitomycin	26	136.0 (34.2-182.5)
mitoxantrone	79	106.0 (16.0-431.5)
nilotinib	93	177.0 (18.0-612.0)
nintedanib	62	131.0 (28.8-314.0)
octreotide	54	323.5 (85.2-1,024.0)
osimertinib	97	123.0 (31.0-272.0)
pazopanib	117	43.0 (20.0-109.0)
ponatinib	42	61.0 (16.2-318.8)
ramucirumab	31	34.0 (21.0-60.0)
ruxolitinib	115	162.0 (49.0-672.5)
selumetinib	6	89.5 (55.0-112.0)
sunitinib	265	69.0 (25.0-202.0)
temsirolimus	29	74.0 (11.0-152.0)
thalidomide	153	55.0 (17.0-172.0)
tisagenlecleucel	7	4.0 (2.5-31.5)
trabectedin	33	35.0 (6.0-103.0)
trametinib	130	94.5 (35.2-206.8)
trastuzumab	1137	158.0 (71.0-297.0)
trastuzumab deruxtecan	27	112.0 (69.5-228.0)
trastuzumab emtansine	62	91.0 (32.5-385.0)
vinblastine	29	16.0 (3.0-104.0)
vinorelbine	97	20.0 (0.0-61.0)

Supplemental Table 7

Cancer indication for the anticancer drugs associated with cancer therapy-related cardiac dysfunction (CTRCD) overreporting in VigiBase® in primary analysis. Date of extraction was March, 1st, 2024.

	Cancer indications																							
	Bone		Breast		Upper aero-digestive tract		Hepatobiliary		Oesophageal		Pancreatic		Stomach		Other digestive		Prostate		Kidney		Other genitourinary		Gynecologic	
Alkylating agents																								
trabectedin	2/83	2.4%	-	-	-	-	-	-	-	-	-	-	-	-	-	-	1/83	1.2%	-	-	-	-	14/83	16.9%
ADT																								
bicalutamide	2/227	0.9%	-	-	3/227	1.3%	-	-	-	-	1/227	0.4%	-	-	-	-	200/227	88.1%	2/227	0.9%	-	-	1/227	0.4%
abiraterone	1/421	0.2%	-	-	-	-	-	-	-	-	-	-	-	-	-	-	416/421	98.8%	-	-	-	-	-	-
Anthracyclines																								
daunorubicin	-	-	3/272	1.1%	-	-	-	-	-	-	-	-	-	-	-	-	-	-	-	-	-	-	-	-
doxorubicin	65/1,486	4.4%	576/1,486	38.8%	2/1,486	0.1%	12/1,486	0.8%	2/1,486	0.1%	2/1,486	0.1%	2/1,486	0.1%	6/1,486	0.4%	6/1,486	0.4%	5/1,486	0.3%	1/1,486	0.1%	98/1,486	6.6%
epirubicin	1/486	0.2%	458/486	94.2%	-	-	-	-	1/486	0.2%	1/486	0.2%	7/486	1.4%	-	-	-	-	1/486	0.2%	2/486	0.4%	-	-
mitoxantrone	1/154	0.6%	25/154	16.2%	-	-	1/154	0.6%	-	-	-	-	-	-	-	-	13/154	8.4%	-	-	-	-	-	-
Antimetabolites																								
clofarabine	-	-	-	-	-	-	-	-	-	-	-	-	-	-	-	-	-	-	-	-	-	-	-	-
fludarabine	-	-	1/164	0.6%	-	-	1/164	0.6%	-	-	-	-	1/164	0.6%	-	-	-	-	-	-	-	-	1/164	0.6%
Immunomodulating agents																								
thalidomide	1/653	0.2%	1/653	0.2%	2/653	0.3%	-	-	-	-	-	-	-	-	3/653	0.5%	12/653	1.8%	13/653	2.0%	-	-	5/653	0.8%
Kinase inhibitors																								
bosutinib	-	-	1/171	0.6%	-	-	-	-	-	-	-	-	-	-	-	-	-	-	-	-	-	-	-	-
dasatinib	1/944	0.1%	-	-	-	-	-	-	-	-	-	-	-	-	-	-	6/944	0.6%	1/944	0.1%	-	-	-	-
imatinib	-	-	3/609	0.5%	-	-	1/609	0.2%	-	-	-	-	3/609	0.5%	4/609	0.7%	4/609	0.7%	4/609	0.7%	-	-	3/609	0.5%
ponatinib	-	-	-	-	-	-	-	-	-	-	-	-	-	-	1/184	0.5%	-	-	-	-	-	-	-	-
ibrutinib	-	-	1/1,067	0.1%	-	-	-	-	-	-	-	-	-	-	-	-	4/1,067	0.4%	-	-	-	-	-	-
osimertinib	-	-	1/245	0.4%	-	-	-	-	-	-	-	-	-	-	-	-	1/245	0.4%	-	-	-	-	-	-
trametinib	-	-	-	-	-	-	1/295	0.3%	-	-	1/295	0.3%	-	-	3/295	1.0%	1/295	0.3%	1/295	0.3%	-	-	4/295	1.4%
entrectinib	-	-	-	-	-	-	1/24	4.2%	1/24	4.2%	-	-	-	-	1/24	4.2%	-	-	-	-	-	-	-	-
sunitinib	3/599	0.5%	19/599	3.2%	-	-	1/599	0.2%	1/599	0.2%	6/599	1.0%	2/599	0.3%	3/599	0.5%	6/599	1.0%	504/599	84.1%	-	-	1/599	0.2%
Monoclonal antibodies																								
gemtuzumab ozogamicin	-	-	1/72	1.4%	-	-	-	-	-	-	-	-	-	-	-	-	-	-	-	-	-	-	-	-
trastuzumab	-	-	2,464/2,542	96.9%	7/2,542	0.3%	2/2,542	0.1%	4/2,542	0.2%	-	-	28/2,542	1.1%	3/2,542	0.1%	3/2,542	0.1%	2/2,542	0.1%	-	-	5/2,542	0.2%
trastuzumab emtansine	-	-	143/151	94.7%	2/151	1.3%	-	-	-	-	-	-	-	-	-	-	2/151	1.3%	-	-	-	-	1/151	0.7%
Proteasom inhibitors																								
bortezomib	1/1,052	0.1%	1/1,052	0.1%	1/1,052	0.1%	-	-	-	-	1/1,052	0.1%	-	-	1/1,052	0.1%	3/1,052	0.3%	2/1,052	0.2%	-	-	-	-
carfilzomib	-	-	2/802	0.2%	-	-	-	-	-	-	-	-	-	-	-	-	1/802	0.1%	-	-	-	-	-	-
Others																								
anagrelide	-	-	2/187	1.1%	-	-	-	-	-	-	-	-	-	-	-	-	1/187	0.5%	-	-	-	-	-	-

	Cancer indications																					
	Leukaemia		Lymphoma		Myeloma		Other haematologic		Lung or pleural		Thymoma		Nervous system		Melanoma		Other skin		Soft tissues		Endocrine	
**Alkylating agents**																						
trabectedin	-	-	-	-	-	-	-	-	-	-	-	-	-	-	-	-	-	-	65/83	78.3%	1/83	1.2%
**ADT**																						
bicalutamide	2/227	0.9%	1/227	0.4%	1/227	0.4%	3/227	1.3%	3/227	1.3%	-	-	-	-	-	-	1/227	0.4%	-	-	1/227	0.4%
abiraterone	-	-	-	-	1/421	0.2%	-	-	-	-	-	-	-	-	-	-	-	-	-	-	-	-
**Anthracyclines**																						
daunorubicin	269/272	98.9%	2/272	0.7%	-	-	1/272	0.4%	-	-	-	-	-	-	-	-	-	-	-	-	-	-
doxorubicin	76/1,486	5.1%	308/1,486	20.7%	143/1,486	9.6%	10/1,486	0.7%	6/1,486	0.4%	4/1,486	0.3%	23/1,486	1.5%	3/1,486	0.2%	1/1,486	0.1%	116/1,486	7.8%	11/1,486	0.7%
epirubicin	3/486	0.6%	2/486	0.4%	1/486	0.2%	-	-	3/486	0.6%	-	-	1/486	0.2%	-	-	-	-	10/486	2.1%	-	-
mitoxantrone	101/154	65.6%	7/154	4.5%	2/154	1.3%	1/154	0.6%	-	-	-	-	-	-	1/154	0.6%	-	-	3/154	1.9%	-	-
**Antimetabolites**																						
clofarabine	86/93	92.5%	1/93	1.1%	-	-	7/93	7.5%	-	-	-	-	-	-	-	-	-	-	-	-	-	-
fludarabine	112/164	68.3%	18/164	11.0%	7/164	4.3%	14/164	8.5%	3/164	1.8%	-	-	1/164	0.6%	7/164	4.3%	-	-	-	-	-	-
**Immunomodulating agents**																						
thalidomide	6/653	0.9%	4/653	0.6%	505/653	77.3%	81/653	12.4%	3/653	0.5%	-	-	2/653	0.3%	11/653	1.7%	-	-	2/653	0.3%	-	-
**Kinase inhibitors**																						
bosutinib	168/171	98.2%	1/171	0.6%	-	-	1/171	0.6%	-	-	-	-	-	-	-	-	-	-	1/171	0.6%	-	-
dasatinib	924/944	97.9%	4/944	0.4%	1/944	0.1%	3/944	0.3%	6/944	0.6%	-	-	-	-	3/944	0.3%	-	-	1/944	0.1%	-	-
imatinib	561/609	92.1%	4/609	0.7%	3/609	0.5%	7/609	1.1%	3/609	0.5%	-	-	1/609	0.2%	1/609	0.2%	-	-	7/609	1.1%	1/609	0.2%
ponatinib	184/184	100.0%	-	-	-	-	-	-	-	-	-	-	-	-	-	-	-	-	-	-	-	-
ibrutinib	732/1,067	68.6%	341/1,067	32.0%	4/1,067	0.4%	2/1,067	0.2%	1/1,067	0.1%	-	-	-	-	1/1,067	0.1%	2/1,067	0.2%	-	-	-	-
osimertinib	-	-	-	-	-	-	-	-	241/245	98.4%	-	-	-	-	-	-	1/245	0.4%	-	-	-	-
trametinib	2/295	0.7%	1/295	0.3%	4/295	1.4%	-	-	27/295	9.2%	-	-	11/295	3.7%	226/295	76.6%	-	-	6/295	2.0%	5/295	1.7%
entrectinib	-	-	-	-	-	-	-	-	21/24	87.5%	-	-	-	-	-	-	-	-	-	-	1/24	4.2%
sunitinib	3/599	0.5%	1/599	0.2%	-	-	2/599	0.3%	6/599	1.0%	7/599	1.2%	-	-	1/599	0.2%	-	-	5/599	0.8%	29/599	4.8%
**Monoclonal antibodies**																						
gemtuzumab ozogamicin	72/72	100.0%	1/72	1.4%	-	-	-	-	-	-	-	-	-	-	-	-	-	-	-	-	-	-
trastuzumab	3/2,542	0.1%	3/2,542	0.1%	3/2,542	0.1%	1/2,542	0.0%	5/2,542	0.2%	-	-	1/2,542	0.0%	-	-	-	-	1/2,542	0.0%	-	-
trastuzumab emtansine	-	-	-	-	-	-	-	-	-	-	-	-	1/151	0.7%	-	-	-	-	-	-	-	-
**Proteasom inhibitors**																						
bortezomib	40/1,052	3.8%	37/1,052	3.5%	962/1,052	91.4%	3/1,052	0.3%	9/1,052	0.9%	-	-	1/1,052	0.1%	-	-	-	-	-	-	-	-
carfilzomib	4/802	0.5%	2/802	0.2%	793/802	98.9%	-	-	-	-	-	-	-	-	-	-	-	-	-	-	2/802	0.2%
**Others**																						
anagrelide	3/187	1.6%	-	-	-	-	180/187	96.3%	-	-	-	-	-	-	-	-	1/187	0.5%	-	-	-	-

Supplemental Figure 1

Associations between CTRCD reporting and concurrent medications, concurrent reactions, and demographic characteristics of patients in VigiBase® in the primary analysis population (anticancer drug population). Data in VigiBase® were extracted on March, 1st, 2024. A total of 42,828 CTRCD cases, and a total of 4,110,900 anticancer adverse drug reaction reports in the overall database were found at that time. Concomitant HF drugs were used as proxies to history of HF. RAAS: renin angiotensin aldosterone system, ROR: reporting odd-ratio.

Condtions	ROR (95% CI)
Age, years	
<18 (ref)	1.00 (1.00–1.00)
18–45	0.86 (0.79–0.94)
45–65	1.11 (1.02–1.20)
65–75	1.58 (1.46–1.71)
>75	2.20 (2.03–2.39)
Sex, male	1.08 (1.05–1.11)
Concomitant drugs	
Agents acting on the RAAS	1.13 (1.09–1.18)
Beta–blocking agents	1.50 (1.44–1.56)
Diuretics	2.40 (2.31–2.49)
Concomitant situations	
Acute coronary syndrome	4.01 (3.77–4.27)
Cardiac conduction disorders	2.38 (2.05–2.77)
Cardiac valve disorders	12.39 (11.19–13.72)
Embolic and thrombotic venous events	1.64 (1.53–1.76)
Hypertension	1.62 (1.53–1.72)
Hyperthyroidism	1.06 (0.79–1.42)
Myocarditis	9.21 (7.95–10.67)
Supraventricular tachyarrhthmias	5.79 (5.48–6.11)
Torsade de pointes/QT prolongation	1.23 (1.02–1.48)
Ventricular tachyarrhythmias	0.67 (0.58–0.80)

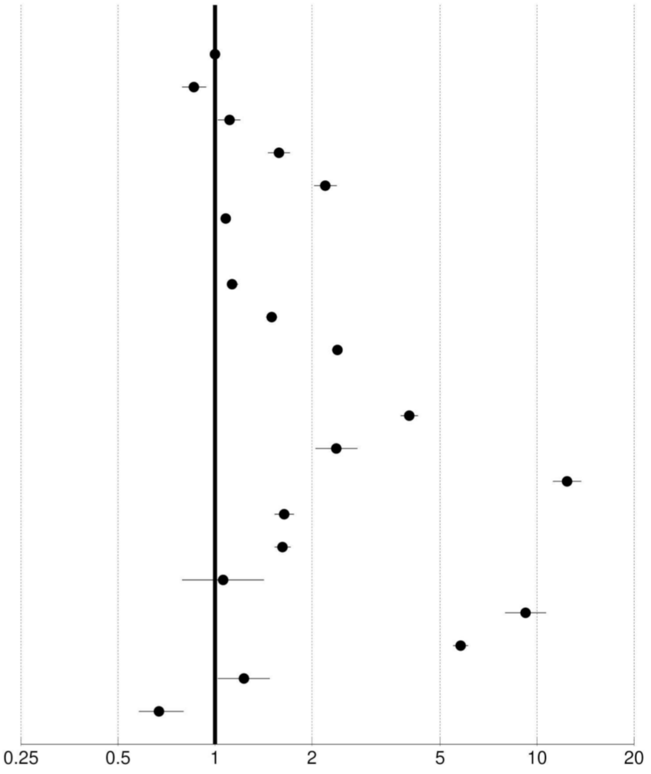

Supplemental Figure 2

Overview of the most prevalent anticancer drug regimens among cancer therapy-related cardiac dysfunction (CTRCD) reports in Vigibase®. Anticancer drugs associated with CTRCD in the primary analysis are highlighted in red. Date of extraction was March, 1st, 2024.

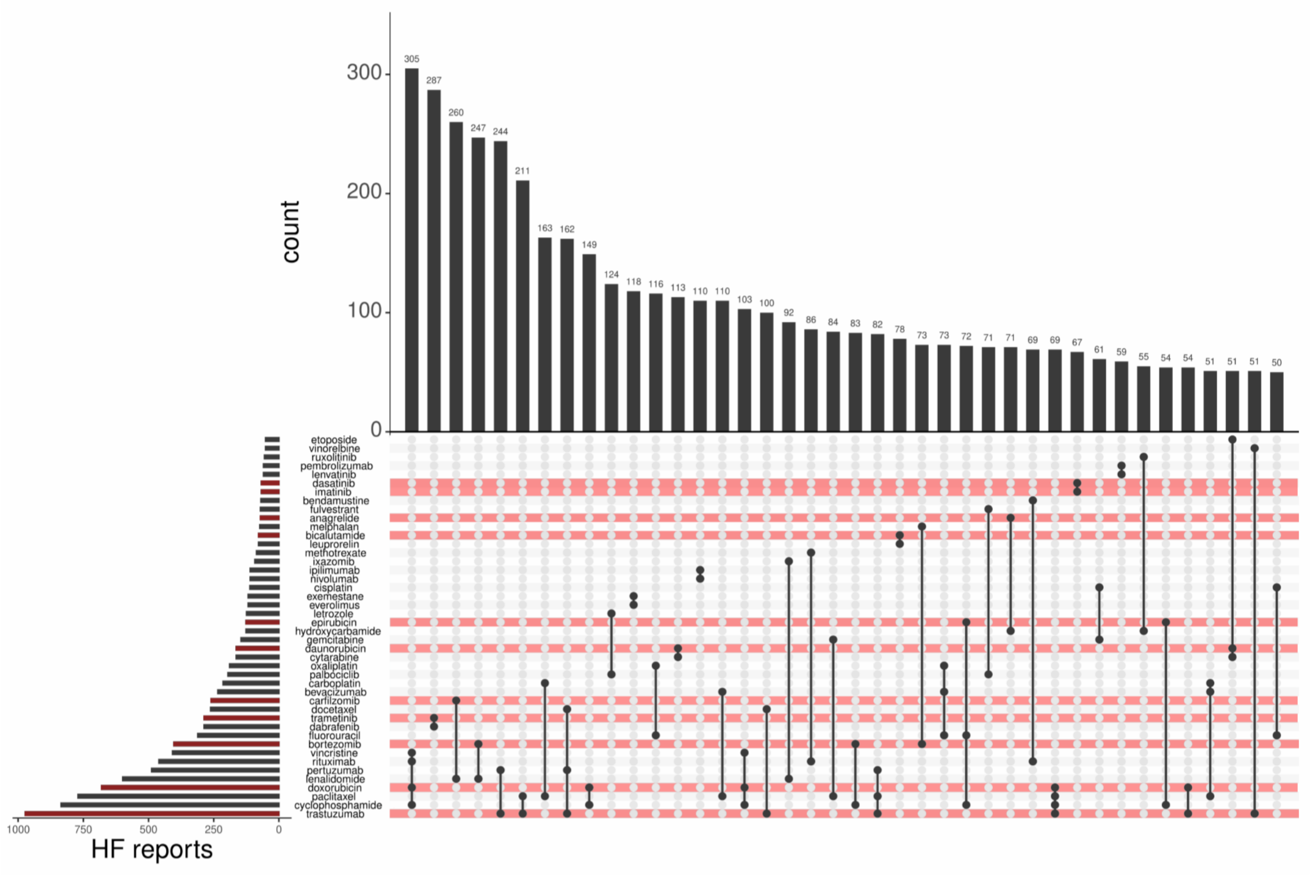

Supplemental Figure 3

Associations between cancer therapy-related cardiac dysfunction (CTRCD) cases and indications for cancer therapies in VigiBase® within the primary analysis population (anticancer drug population). Univariate analysis. Data from VigiBase® were extracted on March 1st, 2024, comprising a total of 1,838,340 anticancer adverse drug reaction reports with available information about cancer indications in the database at that time.

Cancer indication	n CTRCD reports	total cancer cases	ROR (95% CI)
Bone	116	8785	0.97 (0.80–1.16)
Breast	4800	271357	1.37 (1.33–1.42)
Upper aerodigestive tract	90	12448	0.52 (0.43–0.65)
Hepatobiliary	107	18390	0.42 (0.35–0.51)
Oesophageal	60	8568	0.51 (0.39–0.66)
Pancreatic	291	27689	0.76 (0.68–0.86)
Stomach	110	18730	0.42 (0.35–0.51)
Other digestive	778	130401	0.42 (0.39–0.45)
Gynecologic	537	80457	0.47 (0.44–0.52)
Prostate	1519	115599	0.96 (0.91–1.01)
Kidney	1374	84971	1.20 (1.13–1.27)
Other genitourinary	193	21366	0.66 (0.57–0.76)
Leukaemia	4155	203354	1.61 (1.55–1.66)
Lymphoma	1104	67641	1.21 (1.14–1.28)
Myeloma	5164	396812	0.94 (0.91–0.97)
Other haemaologic	1447	79985	1.35 (1.28–1.43)
Lung or pleural	1763	153837	0.83 (0.79–0.87)
Thymoma	19	1157	1.21 (0.77–1.90)
Nervous system	152	29568	0.37 (0.31–0.43)
Ocular	6	1782	0.24 (0.11–0.54)
Melanoma	599	52165	0.84 (0.77–0.91)
Other skin	76	10479	0.53 (0.42–0.66)
Soft tissue	327	14915	1.63 (1.46–1.82)
Endocrine	418	34680	0.88 (0.80–0.97)

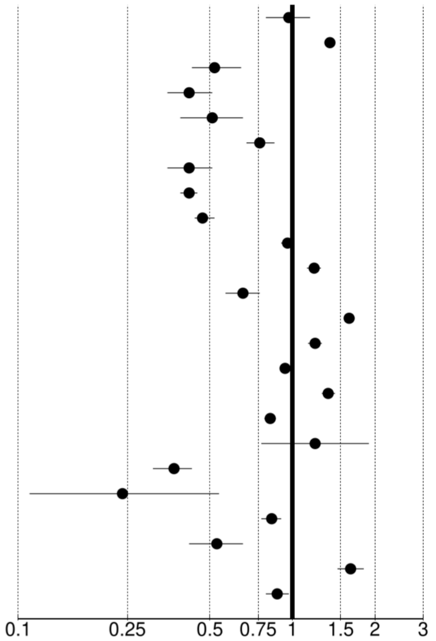

Supplement: pvaf027_Supplementary_Data [file pvaf027_supplementary_data.pdf]
